# Repurposing of FDA approved ring systems through bi-directional target-ring system dual screening

**DOI:** 10.1038/s41598-020-78077-9

**Published:** 2020-12-03

**Authors:** Surendra Kumar, Cheongyun Jang, Lalita Subedi, Sun Yeou Kim, Mi-hyun Kim

**Affiliations:** Gachon Institute of Pharmaceutical Science and Department of Pharmacy, College of Pharmacy, Gachon University, 191 Hambakmoeiro, Yeonsu-gu, Inchon, Republic of Korea

**Keywords:** Drug discovery, Drug screening, Biological techniques, High-throughput screening, Cheminformatics, Medicinal chemistry

## Abstract

In drug repurposing approaches, the chemically diverse and potentially safe molecules can be explored as therapeutic potential beyond those originally targeted indications. However, accessible information on a limited number of drug pipelines can lead to competitive over-heating issues, and intellectual property rights also restrict the free investigation in chemical space. As a complementary approach to the drawbacks, ring systems of approved drugs (instead of clinical drugs) can be optimized and used for repurposing purposes. In this study, bi-directional target (T) and ring system (R) dual screening (TR screening) was developed for the repurposing of their rarely used ring systems from FDA approved drugs. The TR screening suggested RAR β and cyproheptadine as the best pair of target and ring system to escape a saddle point. The selected ring system was virtually grown and elaborated with the defined criteria: synthesizability, drug-likeness, and docking pose showing the top scores. The achieved compounds were synthesized and biologically tested with an acceptable ADME/T profile.

## Introduction

The current paradigm of drug R&D is ‘selection and concentration’ and the current investment of drug R&D is massive. However, it should be not just ‘massive’ but should be ‘efficiently massive’ such as described in the 5R-framework of AstraZeneca^1^. In particular, the ‘Go/No Go’ decision in R&D stages means the willingness to choose a more promising drug pipeline within available resources^2^. However, the strategy can provide neither the satisfactory success rate of drug pipelines nor the opportunity to clarify black box information of the pipelines. When R&D data cannot support assumed proof of concept (POC), these show the limitation in controlling black box e.g., exposure concentration of a drug, off-target effects, and therapeutic window in clinical level^1^. To overcome the productivity crisis, drug repurposing approaches have been developed^3–5^. The chemically diverse and potentially safe molecules can have new therapeutic potential beyond those originally targeted indications. However, accessible information on a limited number of drug pipelines can lead to competitive over-heating issues, and intellectual property rights also restrict the free investigation in chemical space. Those drawbacks of drug repurposing and productivity crisis made us highly motivated in thinking about how to develop more efficient and effective methods. Notably, repurposing of those ring systems (instead of clinical drugs) can be a complementary approach of drug repositioning to overcome these drawbacks.

**Figure 1:**
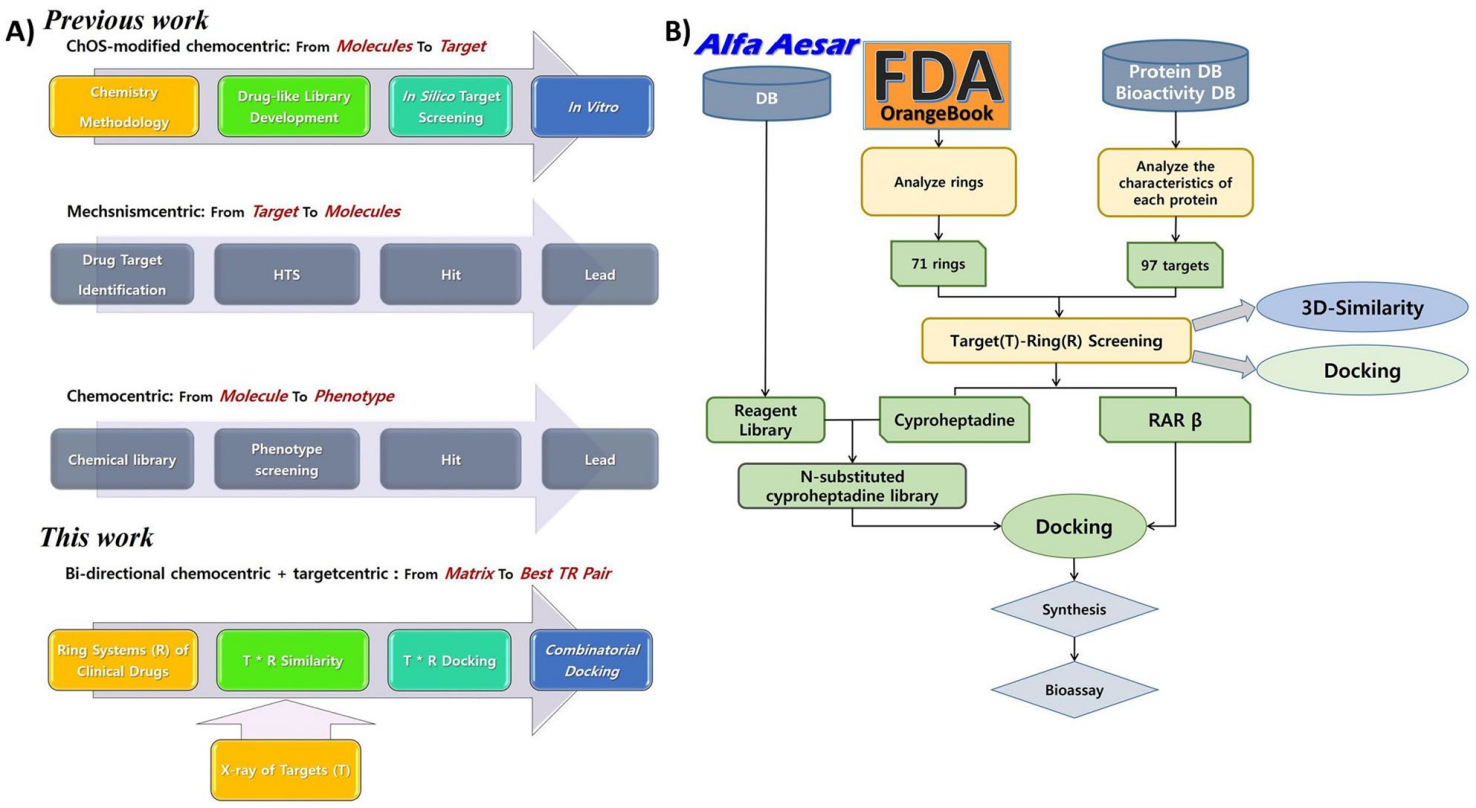
(**A**) Screening strategies in drug discovery and comparison between previous and current work; (**B)** the general workflow for target (T)–ring (R) screening.

Even though any researcher currently cannot suggest a breakthrough paradigm or a magic bullet for drug R&D, accumulated attempts (apart from current approaches) can enable the breakthrough or advance in the future. As an attempt, ‘Chemistry-oriented synthesis (ChOS)’, was proposed by our group^6,7^. The approach is a modified chemo centric screening that considers drug discovery “from an unprecedented drug scaffold” instead of “from a specific target” in Fig. 1A. ChOS approach was designed in the context of screening philosophy, which has been evolved dialectically between ‘drug (chemo centric)’ and ‘target (mechanism centric)’ in Fig. 1A. Remarkably, since the golden age of drug discovery, in the spite of annual approval of around 10 new molecular entities (NMEs) along with the advance of the screening, the stagnation has been observed in developing an unprecedented drug scaffold, a novel drug ring system, and a new ring^8^. As it is, when analyzing frameworks/ring systems/rings of FDA approved drugs, a total of 237 ring systems were rarely used (less than three times) among every ring system in FDA orange book^8^. To further develop the rarely used ring systems, the ChOS approach can respond more efficiently to the stagnation than conventional chemo centric screening. This is because the chemo centric screening requires intensive SAR study with massive synthesis until a novel drug scaffold is achieved. In addition, ChOS approach driven by the novelty of a drug scaffold can be synergized with mechanism centric approach driven by a specific target and can contribute to broadening the drug space of synthetic drugs^7^. However, the ChOS approach cannot develop 237 ring systems simultaneously for the repurposing of such ring systems. Therefore, herein we reported another chemo centric approach modified from the ChOS approach in Fig. 1A. In this study, we established bi-directional target (T) and ring system (R) dual screening for the repurposing, choose best TR pairs from the screening, and combinatorial virtual screening to generate a virtual library with desirable ADMET in Fig. 1B.

## Results

### Descriptor selection for ring system repurposing

For the scalable and bi-directional TR screening, descriptors were assigned to reported ring systems (349) to reduce the number of ring systems. The properties of 349 ring systems extracted from every FDA-approved drug in Orange book^8^ were calculated through CDK toolkit to analyze (1) fragment complexity, (2) hydrogen bond acceptors (HA), (3) hydrogen bond donors (HB), (4) molecular weight, and (5) VABC volume descriptor^9^. Among all the properties calculated, VABC descriptor represents the approximate van der Waals volume (Å^3^/molecule) of molecules and can be calculated by the following formula:

$$ V_{{{\text{vdW}}}} = \sum \,{\text{all}} - {\text{atom}}\,{\text{contributions}} - {5}.{92}N_{{\text{B}}} - {14}.{7}R_{{\text{A}}} - {3}.{8}R_{{{\text{NR}}}} $$

**Figure 2:**
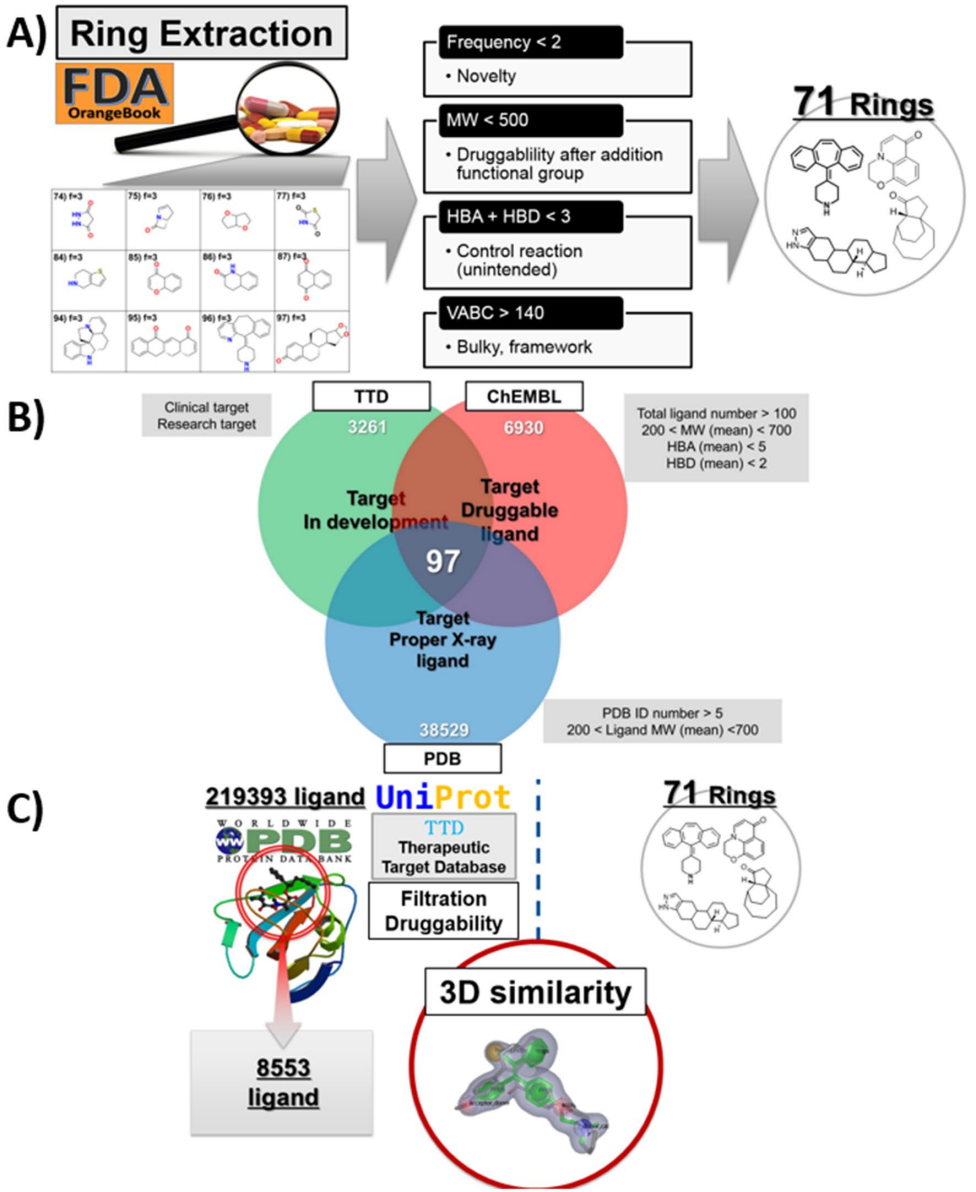
Dimension selection for TR screening. (**A**) Descriptor based selection of ring systems for TR screening; (**B**) target selection for TR screening; (**C**) 3D-similarity (Shape)-based primary TR screening between X-ray ligands and ring systems.

*N*_B_ is the number of bonds, *R*_A_ is the number of aromatic rings, and *R*_NR_ is the number of nonaromatic rings. From 349 ring systems with less than three frequencies in clinically approved drugs, 114 ring systems were chosen based on VABC Volume Descriptor > 140^9^, and 71 ring systems remained after the summation of HA + HB < 3. In this study, the 3D structure of a core (a ring system) rather than side chains (substituents) was focused on maximizing the effect of a ring system on drug–target interaction. According to the criteria mentioned in Fig. 2A, 71 ring systems were selected from a total of 349 ring systems (see supplementary-file 1; Table S1).

### Target selection for repurposing

For the selection of target, from the PDB bank, 38,529 targets were filtered under the conditions of (1) more than five available PDBs of a target, (2) existence of a ligand in the PDBs, (3) carbon construction of a ligand in the PDBs, and (4) desirable range of molecular weight (MW, 250 to 800) to give 1714 targets. The 292 targets were common among TTD database (3261 targets)^10^, ChEMBL version 21 (6930 targets)^11^, and the refined PDB (1714 targets)^12^. After the collection of every ChEMBL ligands from 292 targets, these targets were further manipulated according to the criteria: (1) the number of ligand > 100, (2) MW range 200–700, (3) HBA < 5, and (4) HBD < 2 (Fig. 2B). As a result, 97 targets (no. of PDB: 3424) could be acquired for TR screening.

### Shape-based primary target (T)-ring system (R) screening

Initially, a shape-based calculation between 71 ring systems (as a query) and 3424 ligands of PDBs were performed to produce a 246,512 paired data with several scores from 3 metrics and 2 color/shape (Fig. 2C). Expectedly, the complexity of the bi-direction screening, as well as narrow distribution and deviation of the shape-based scores could not make us choose one ‘target-ring system pair’. In sequence, we selected the *best target against each ring system* (97 rows, see supplementary-file 1; Table S2) and the *best ring system against each target* (71 rows, see supplementary-file 1; Table S3) instead of choosing one uncertain best pair. While TR pairs of the first list, Supplementary Table S2 shows higher similarity (fused score) (0.59–0.84 except for 1 outlier) than TR pairs of the second list, Supplementary Table S3 (0.40–0.84 except for 3 outliers). As an outlier, the pair of the ring (297-302_0) system and target (Uniprot: P19793)^13^ showed 0.99 similarity from the comparison between the core scaffold of Chrysazin and Chrysazin (PDB: 3NSQ). Two targets (Uniprot: P04406 and Q9NUW8) did not have any similar ring system to show two outlier values in the second list, Supplementary Table S3. Because nicotinamide adenine dinucleotide (NAD), the ligand of glyceraldehyde-3-phosphate dehydrogenase is an endogenous cofactor, it showed maximum 0.25 similarity with 71 rings. Another outlier, Spermine, the ligand of tyrosyl-DNA phosphodiesterase 1 (Uniprot: Q9NUW8) does not contain any ring to produce 0 similarity. PDBs annotated in the two best lists (best target, best ring system) were collected and after the annotation of the accumulated count of duplicated PDBs, the duplicate was removed to present 131 PDBs (97 targets) for docking based TR screening.

### Docking based secondary TR screening

**Figure 3:**
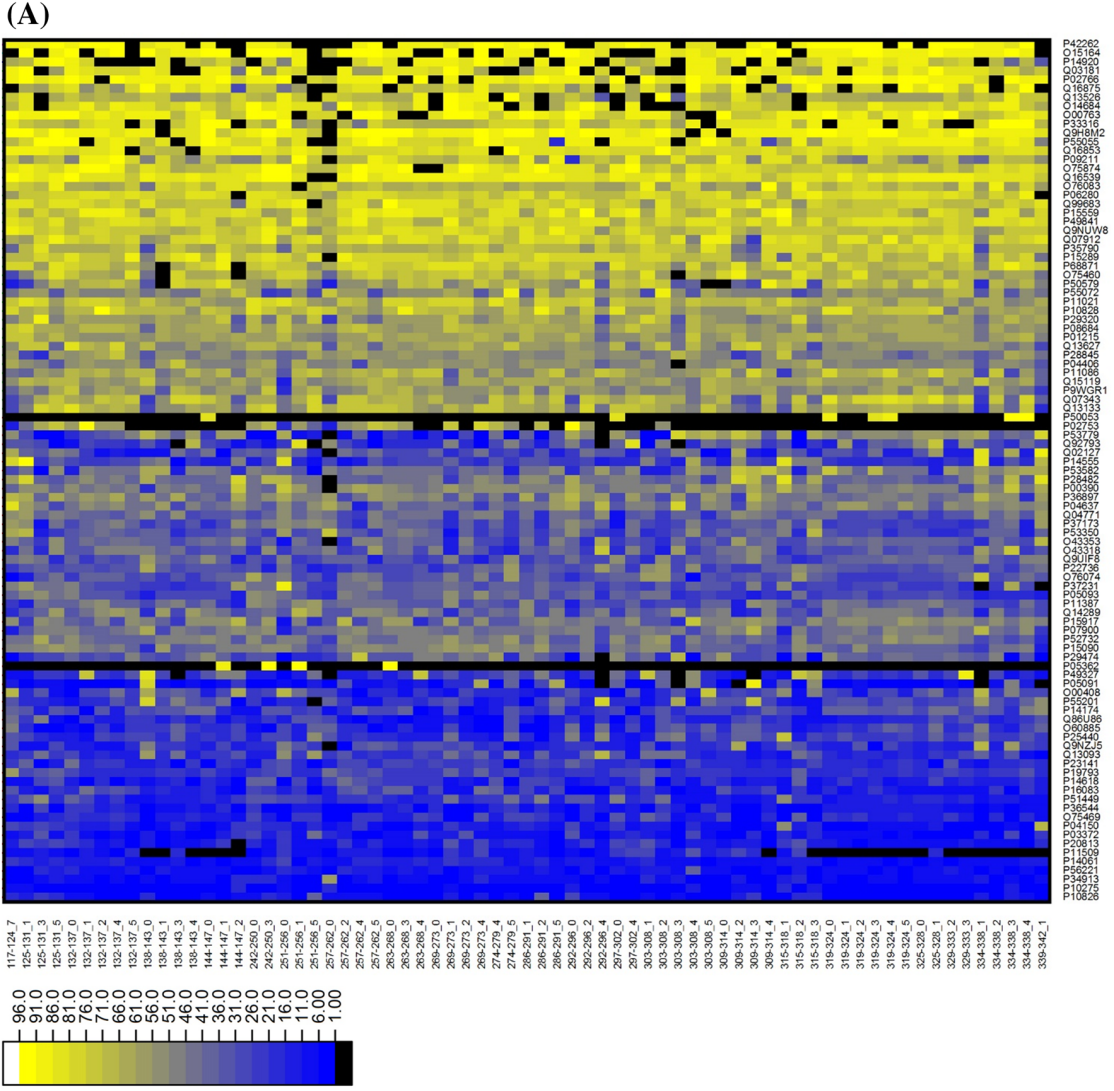

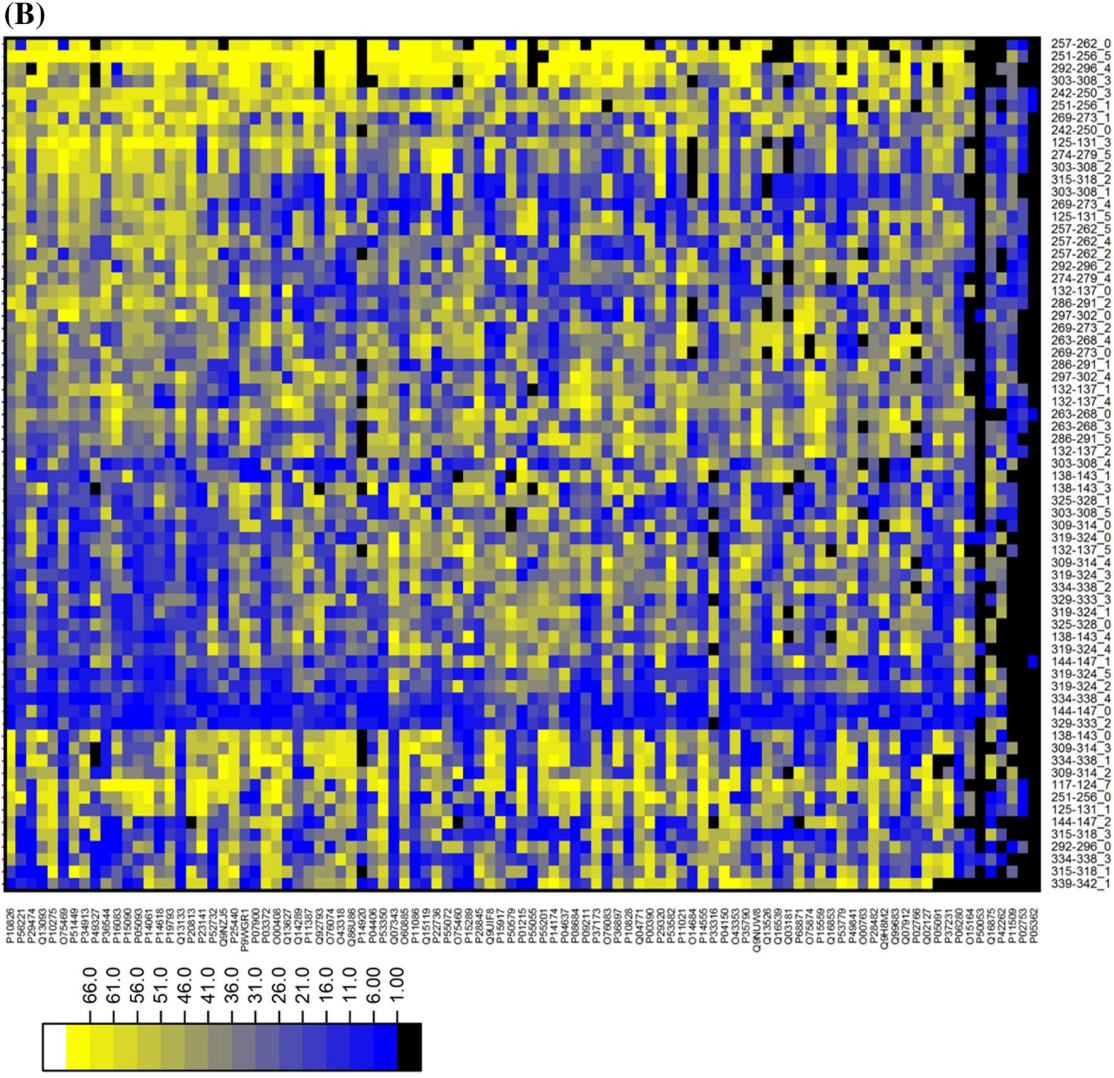
Docking based TR screening result. Heatmap show ranking of TR pairs. (**A**) The ranking of targets (97) against ring systems from docking based TR screening. The x-axis and y-axis represent the Ring ID, Target ID respectively. The ranking of targets are shown by color palette pattern with different color intensity. The higher raking targets are shown as dark blue and lowest raking with yellow color. (**B**) The ranking of ring systems (69) against targets from docking based TR screening. The x-axis and y-axis represent the Ring ID Target ID, respectively. The ranking of ring systems are shown by color palette pattern with different color intensity. The higher raking targets are shown as dark blue and lowest raking with yellow color.

To select the best pair of targets and rings, secondary screening comprised of molecular docking performed on 71 rings (R) system and 131 PDBs [97 targets (T)]. Due to missing atom types for ring (274-279_2, 347-349_2) system, it was excluded during docking. The two search modes were employed in the search for binding mode. After bi-directional docking, the Uniprot accession id assigned to PDBs was used for merging ‘ring-PDB’ into ‘ring-target’ to generate only a single docking result of every TR pair. A matrix of (97 row × 69 columns) was prepared (see supplementary-file 2) that consists of docking score values. These values for each target and each ring were transformed into ranking values and two heatmaps were created from ranked targets (Fig. 3A) and rings (Fig. 3B). The heatmap showed the data in different color intensity with high ranked as dark blue and low with yellow color. Remarkably, three discriminative groups between 97 targets could be observed in the heatmap (Fig. 3A). So, it is helpful to think which targets are more or less reliable for all rings systems. In addition, as shown in the dark blue region area of Fig. 3B, steroid like ring systems such as ‘334-338_4’ (best ring system of 16 targets) and ‘144-147_0’ (best ring system of 12 targets) in Table 1 and Supplementary Table S4 were the highest frequency despite their fragment complexity and alkaloid-like ring systems with amine group followed by the steroid-like. Even though primary TR screening assigned the best target into every ring system, 36 ring systems could not be the best ring of any target after secondary TR screening.

**Table 1:** Docking based secondary TR screening chosen result: best ring system–target pairs.

Ring-ID ^a^	Characteristic (no. of Ring) ^b^	FC^c^	Uniprot ID^d^
334-338_4	Steroid-like (5)	2120.02	Q13093, P51449, P34913, P05093, P20813, P52732, P9WGR1, O43318, P53350, P37173, P36897, Q04771, P04150, O43353, P15559, P28482
144-147_0	Steroid-like (4)	1422.02	P23141, P01215, P00390, P11021, O14684, Q13526, Q16539, O75874, P02766, P37231, O15164, P50053
329-333_2	Steroid-like (4)	1422.02	P10275, P14061, P03372, O76083, P10828, P68871
144-147_2	Steroid-like (5)	1981.02	P56221, P14618, P55201, Q03181, Q99683, Q16875
334-338_3	Alkaloid-like (5)	1469.03	P29474, P10275, P36544, P14920, P11086, P42262
292-296_0	Alkaloid-like (3)	814.02	P49327, P16083, Q14289, P11387, Q07343, P09211
315-318_1	Alkaloid-like (4)	919.02	P07900, P15289, Q16853, P06280
144-147_1	Tetracycline derivative (4)	1141.02	P49841, O00763, Q9H8M2, Q02127
315-318_3	Alkaloid-like (4)	1215.02	Q13627,Q86U86, P50579, P08684
339-342_1	Alkaloid-like (4)	1429.01	P10826, P15090, Q13133
257-262_4	Alkaloid-like (3)	520.01	P04406, P04637, P33316
251-256_0	Alkaloid-like (3)	444.02	Q15119, P35790, P02753
319-324_5	Steroid-like (4)	1719.01	P28845, P05091
257-262_5	Alkaloid-like (3)	628.01	P55072, P29320,
269-273_4	Alkaloid-like (3)	547.02	Q9UIF8, P53779

^a^Ring system ID was assigned according to the literature^7^, ^b^the number of rings consisting of ring systems, ^c^FC: fragment complexity, ^d^Uniprot ID: protein identifier in UniProt protein database.

### Overview of the selected best TR pair

**Figure 4:**
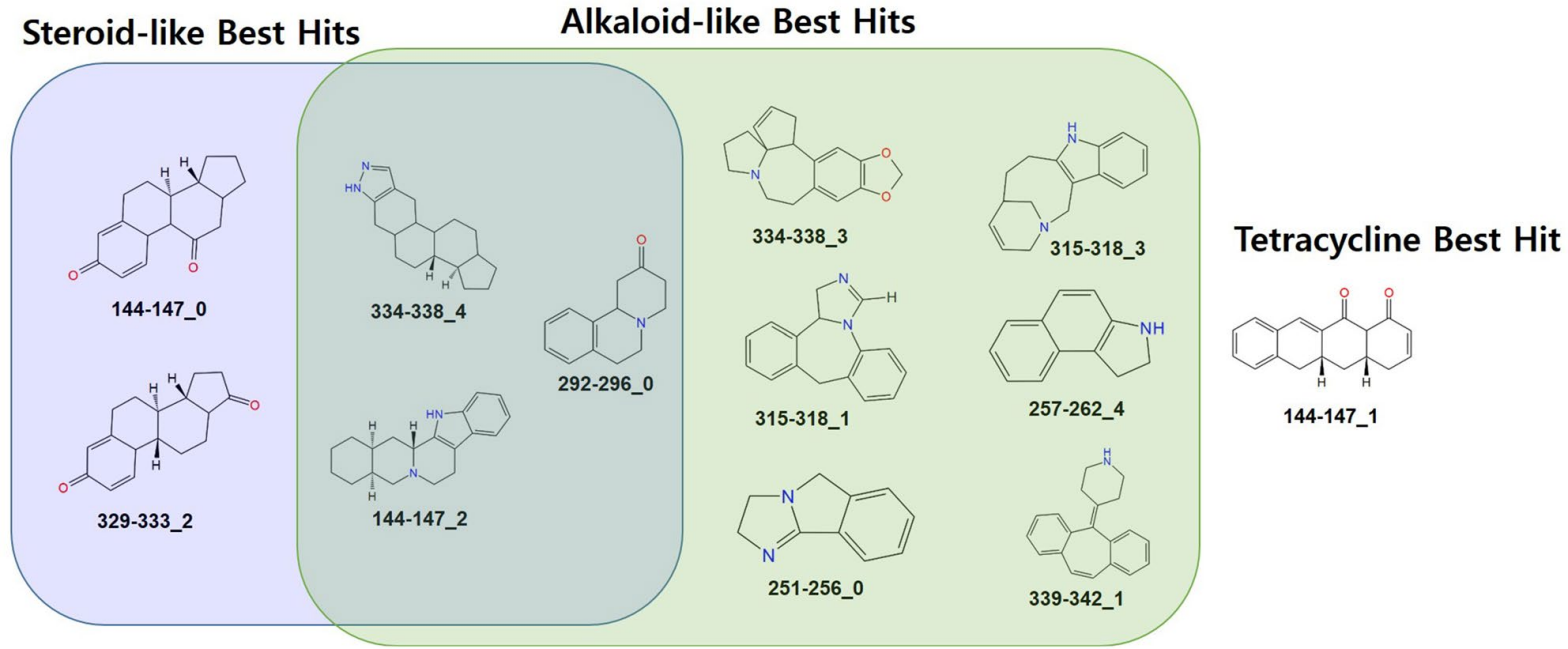
Characterization of frequent TR hits.

In an attempt to experimentally demonstrate best TR pairs from the screening, one pair was chosen according to three criteria: (1) synthetic difficulty and efficiency, (2) readily availability of the commercial source, (3) availability of biological assay, and (4) specificity. Steroid-like ‘334-338_4’ (16 targets) and ‘144-147_0’ (12 targets), privileged ring systems were suspected for their target specificity so that 28 TR pairs were excluded (Table 1, Fig. 4,). In alkaloid-like ring systems (eg. ‘292-296_0’, ‘334-338_3’, ‘315-318_1’, and ‘251-256_0’), late-stage functionalization of their tertiary amines^14–16^ is very challengeable for establishing a substituent in stable C–H bond so that 19 TR pairs of four ring systems were excluded. In contrast to excluded ring systems and their pairs, cyproheptadine (ID: 339-342_1) was readily available so that exactly the ring system, itself is commercially available. While the ring system is rigid without a rotatable bond, the amine group of the ring system facilitates the introduction of functional groups during the optimization. Cyproheptadine is the ring system of loratadine, an approved anti-histamine drug^17^. At the early stage level, it also was reported as an efflux pump inhibitor for mycobacterium tuberculosis^18^, a MAGL inhibitor^19^, an N-type calcium channel inhibitor^20^ but it was never considered for RAR β in the literature study. Recently, cyproheptadine was also reported as an epigenetic modulator, an inhibitor of histone methyltransferase SETD7 with its PDBs (ID: 5AYF, 5YLT)^21^. Even though cyproheptadine existed in the protein database during our screening, it was excluded due to its absence in the TTD database^10^. Notably, cyproheptadine and target (Uniprot ID: P10826) pair could be satisfactory for both target specificity and readily available bioassay. The selected target belongs to the family of nuclear receptor denoted as RAR β (Retinoid Acid Receptor Beta). The natural modular of this receptor is retinoic acid, which specifically binds to the retinoic acid receptor and then forms heterodimers with other receptors such as estrogen receptor alpha and AP-1 receptor and further activates downstream to regulate cell differentiation^14,22^. The retinoic acid receptor is divided into three classes of alpha, beta, and gamma, of which beta has been shown to have anti-cancer effects in epithelial cells. Multiple studies reported that RARβ plays a role in the breast cancer cell metastatic process and found to interact with ATRA (all-trans-retinoic acid)^23^. Additionally, the neuroprotective effect of retinoic acid, as well as its anti-cancer effect, is now being revealed^24^.

### Combinatorial virtual screening of RAR beta and cyproheptadine as a chosen TR pair

**Figure 5:**
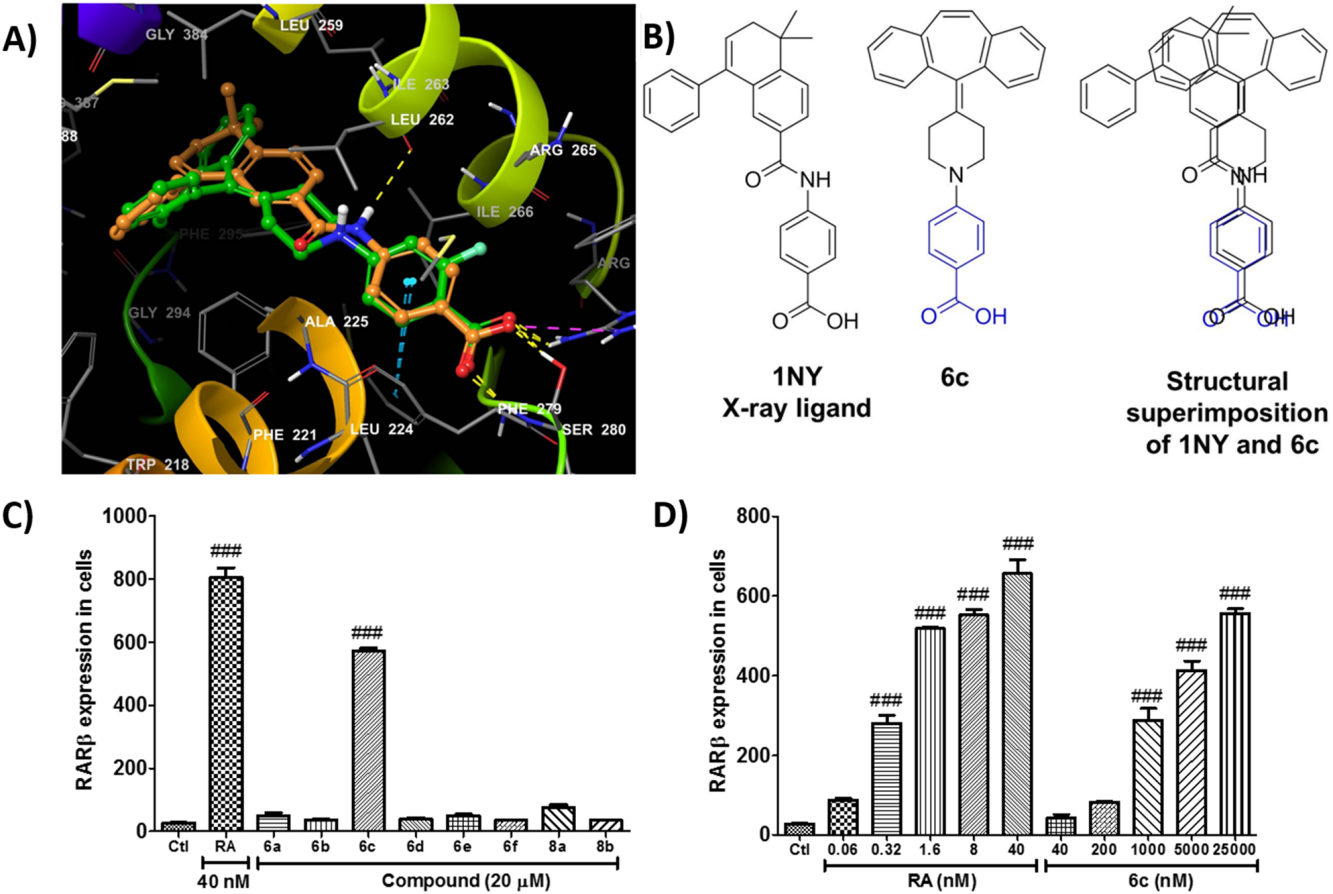
(**A**) The binding mode and superimposition of docked X-ray ligand and 6c; (**B**) the structural superimposition of X-ray ligand and 6c; (**C**) the human RAR β reporter bioassay for synthesized compound, (**D**) the concentration dependent RAR β bioassay for RA (all trans retinoic acid) and 6c.

Since the X-ray ligand in RAR β (PDB: 4JYG) has a carboxyl group and establishes H-bond with Ser280 and Arg269 residues of the retinoic acid receptor, thus virtual reagents with the functional group were generated based on commercial available reagents, selected, and used in creation of the virtual libraries to mimic the interactions. During the virtual library generation, the cyproheptadine was set to core structure in demethylation state, and libraries were grown to amine position. Additionally, the druggability of every compound in the virtual library was predicted to filter out undesirable compounds according to the pre-defined criteria mentioned in the experiments and methods section and a total of 4954 compounds were retained. These compounds were further docked into the selected target protein (PDB ID: 4JYG) to rank them. During docking, the energy window for ring sampling was increased to enhance the conformer sampling and write out at most 2000 poses per ligand. From all the docking results, the poses were ranked according to docking scores, and top-scoring poses selected for interaction analysis. Comparing the binding mode of top-ranked poses from the virtual library and crystal bound ligand, we found a structural complementary in their binding mode (Fig. 5A). Among all the poses selected, compound 6c proposed maximum binding affinity in terms of docking score of − 15.568 kcal/mol. Similarly, the compound 6b followed the compound 6c with a score of − 15.324 kcal/mol (Table 2). In addition, compound 8a, 6d, 6e, 6a, shows lower docking score and 6f shows the lowest among selected poses. Also, chosen compounds showed acceptable ADMET properties in the prediction of solubility, polar surface area, albumin binding, BBB penetration, hERG inhibition, cell permeability (Caco-2 and MDCK), and CNS activity (Table 3). While their aqueous solubility (logS) is slightly lower than the standard range, their cell permeability indicates desirable concern in active transport as well as simple diffusion relying on lipophilicity. In addition, every compound passed PAINS (pan assay interference compound) filter and cardiotoxicity filter^25^. The tertiary amine group of virtual compounds contributed to CNS activity and BBB permeability.

**Table 2:** The synthesis of *N*-arylated cyproheptadinederivatives.


Compound ID	R_1_	R_2_	R_3_	R_4_	R_5_	Docking score	Ligand efficiency
6a	H	COOH	CH_3_	H	H	− 11.506	− 0.371
6b	H	CH_3_	COOH	H	H	− 15.324	− 0.494
6c	H	H	COOH	H	H	− 15.568	− 0.519
6d	NO_2_	H	COOH	H	H	− 12.220	− 0.370
6e	H	COOH	H	H	H	− 11.569	− 0.386
6f	COOH	H	H	H	H	− 8.404	− 0.280
8a	NH_2_	H	COOH	H	H	− 12.994	− 0.419
8b	H	NH_2_	H	COOH	H	− 14.352	− 0.463

**Table 3:** ADMET properties of testing compounds.

Compound ID	CNS	logS	logHERG	Apparent Caco-2 permeability (nm/s)	logBB	Apparent MDCK permeability (nm/s)	logKhsa	PSA	PAINS
6a	− 1	− 7.49	− 4.87	325.32	− 0.47	186.92	1.47	49.38	Pass
6b	− 1	− 7.49	− 4.69	323.17	− 0.45	185.58	1.39	50.42	Pass
6c	− 1	− 7.20	− 4.85	252.20	− 0.56	141.95	1.26	52.54	Pass
6d	− 2	− 7.73	− 4.70	62.07	− 1.27	31.19	1.16	89.28	Pass
6e	− 1	− 7.20	− 4.87	248.47	− 0.57	139.68	1.26	53.91	Pass
6f	− 1	− 7.20	− 4.86	441.00	− 0.29	259.70	1.19	44.68	Pass
8a	− 1	− 7.35	− 6.67	708.65	− 0.82	340.94	1.39	64.36	Pass
8b	− 1	− 7.35	− 6.99	731.60	− 0.87	352.90	1.48	66.46	Pass
Std. range	(− 2 Inactive, + 2 active)	(− 6.5 to 0.5)	Concern below -5	< 25 poor, > 500 great	(− 3.0 to 1.2)	< 25 poor, > 500 great	(− 1.5 to 1.5)	(7.0–200)	

The calculated values based on 95% of known drugs from Qikprop v6.2 (Schrodinger Suite 2019-4).

*CNS* predicted central nervous system activity, *logs* conformation-independent predicted aqueous solubility, *logHERG* predicted IC50 value for blockage of HERG K+ channels, *Caco-2* predicted gut-blood barrier permeability, *logBB* predicted brain/blood partition coefficient, *MDCK* predicted apparent MDCK cell permeability, *logKHSA* prediction of binding to human serum albumin, *PSA* Van der Waals surface area of polar nitrogen and oxygen atoms and carbonyl carbon atoms, *PAINS* pain assay interference compounds calculated from RDKit package.

### Synthesis of cyproheptadine derivatives and RAR β agonistic activity

**Figure 6:**
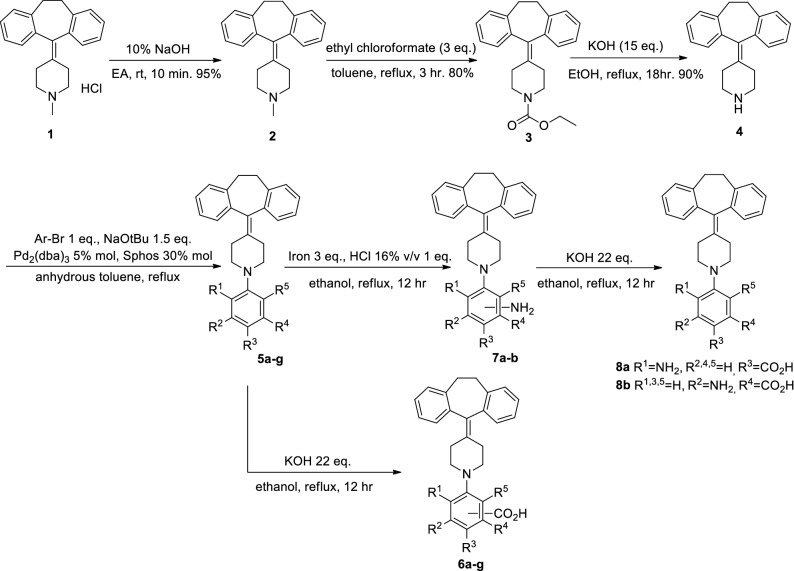
Synthetic scheme of testing compounds.

Based on combinatorial virtual screening, promising compounds were selected for RAR β agonist activity and synthesized (Fig. 6). In the virtual library, the derivatization of phenyl ring at N-1 of cyproheptadine core presented high predicted affinity as well as facile synthetic accessibility, applicable to late-stage functionalization. In contrast to the in silico combinatorial chemistry, additional synthetic steps were required including *N*-demethylation of piperidine ring, deprotection of carbamate group, and saponification of methyl ester. Pd-mediated Buchwald-Hartwig coupling reaction between compound 4 and aryl halides presented moderate yield to show the gap between virtual chemistry and experimental chemistry^26^. The detailed procedure for the synthesis of testing compounds 6a–f and 8a–b was given in Fig. 6 and Supplementary-file 1. The synthesized compounds were further tested for human RAR β transcriptional agonistic activity through cell-based reporter assay. Detected response unit of luciferase, a reporter gene assigned the relative transcriptional activity of RAR β induced by testing compounds. In other words, the detected luminescence means binding of an agonist or antagonist to the ligand-binding domain of RAR β and the triggering of a functional response. Remarkably, the substitution of –COOH group at the R3 position was critical for RAR β agonist activity. If we compare compound 6c (R1=H), 6b (R2=CH_3_), 6d (R1=NO_2_), and 8a (R1=NH_2_), all have docking scores of − 15.57 kcal/mol, − 15.32 kcal/mol, − 12.22 kcal/mol, and − 12.99 kcal/mol respectively, but were not proportion to experimental activity (Fig. 5B). Among tested 8 compounds, compound 6c showed the most promising agonistic activity to prove docking prediction in Fig. 5C. In addition, the compound 6c showed concentration-dependence such as positive control compound, retinoic acid in Fig. 5D.

## Discussion

### Rationale and criteria of TR screening

The selection process for TR screening essentially requires decision criteria on the desired dimensions of the TR matrix. For the purpose, the number of Targets (T) could be decided based on the availability and effectiveness of target data. In addition, the used three databases could provide three criteria, (1) druggability of targets (PDB)^12^, (2) clinical value of targets (TTD)^10^, and (3) availability on known ligands (ChEMBL)^11^. The predefined filtering through the criteria could retain 97 targets (3424 PDBs) covering a wide range of therapeutic targets. The selection included every protein target class such as receptors (eg. GPCR, NR), ion channels, enzymes, and inflammatory factors. In sequence, the number of Ring systems (R) could be decided based on molecular descriptors. While criteria such as HA, HB, and molecular weight can guarantee drug-likeness to permit the introduction of diverse substitution into ring systems, fragment complexity and VABC implied different meanings. VABC, Volume Descriptor > 140 indicates that a ring system consists of six or more carbons^9^ to prevent the ring system from being smaller than its substituents (inverse between the core scaffold and side-chain). Fragment complexity can indicate how much difficult to synthesize derivatives of a chosen ring system. As shown in Supplementary Table S1, fragment complexity as a criterion can make our bi-directional screening discard many ring systems so that VABC > 140 (larger than benzene ring), HA, and HB were only primary criteria for ring selection and other descriptors were considered as secondary criteria at the stage of virtual library generation. Very fortunately, selected 71 rings were diverse in nature ranges from two to six fused rings. These fused ring systems open the wide possibility for site modification. However, synthetic accessibility also should be added to the secondary criteria for further guidance in optimization within the domain of application.

### Opportunity and scope of bi-directional TR screening

**Figure 7:**
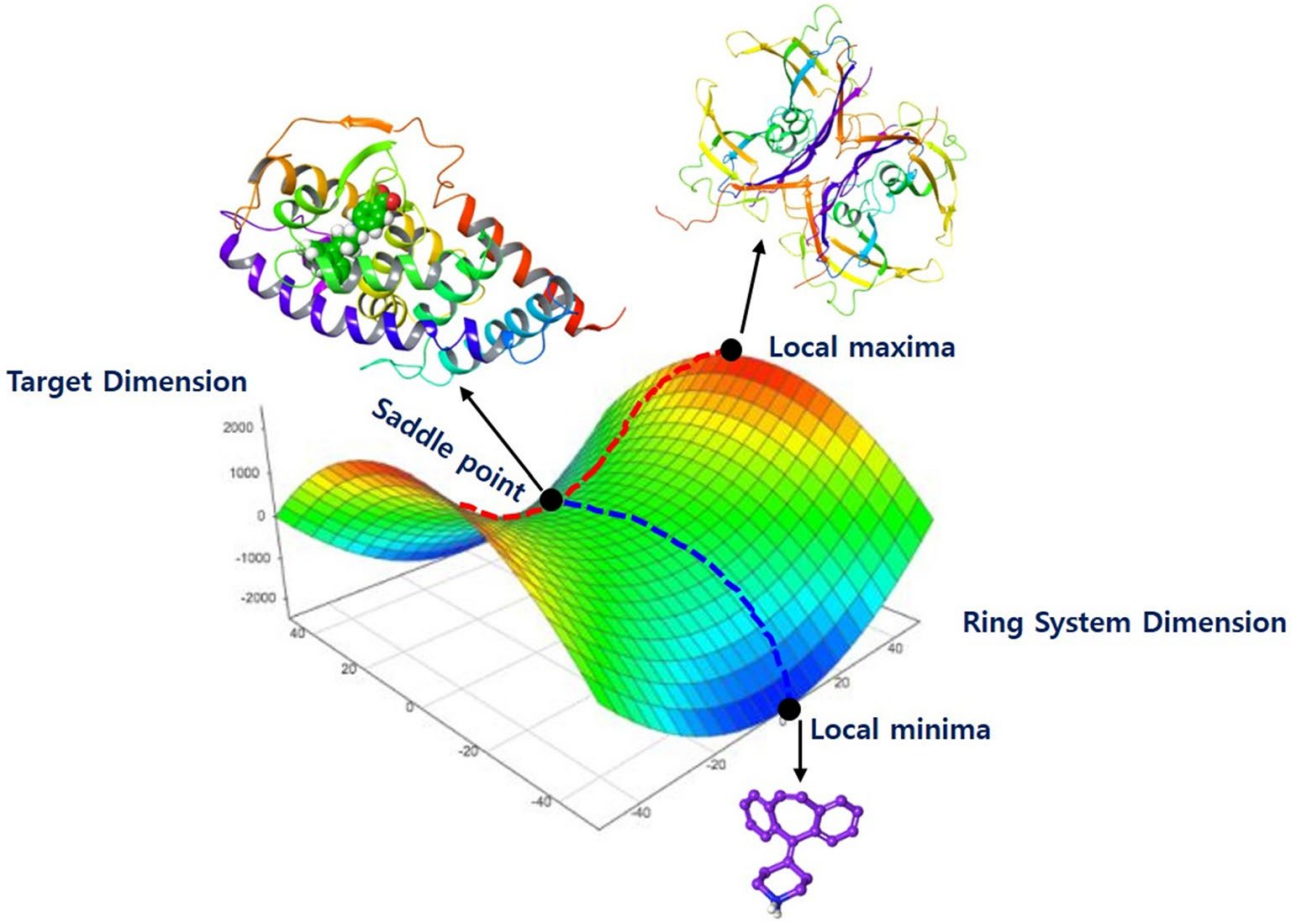
Saddle point in target (T)–ring (R) dimensions.

Rings or ring systems play an important role in medicinal chemistry or combinatorial chemistry, where ring systems are used as central scaffolds or in scaffold hopping where the central core is replaced with a bioisosteric ring system. Ring systems contribute to approximately 97% of bioactive molecules and aliphatic rings also contribute to more than 65%. Almost 73.3% of bioactive molecules have fused aromatic ring systems ranging from one to three simple five or six-membered rings^27^. Therefore, the rarely used ring systems of approved drugs can provide a new repurposing approach distinct from current drug repurposing and also complementary to it. Bi-directional TR dual screening method was designed for the repurposing of rarely used ring systems and embodied through the architecture consisting of multi-dimensional shape-based calculation, structure-based virtual screening, and in silico combinatorial docking for virtual optimization. The architecture of our TR screening reflects the dialectical history of screening approaches (phenotype-based versus mechanism-based). Initially, drug screening was a chemocentric approach based on phenotype due to insufficient understanding of targets and their mechanism. Such chemocentric approach was transferred to molecular biology centric approach based on targets and their mechanism. Philosophy of the new approach was ‘superbly selective *single compound* for a *single target*’ with a known mechanism. Even though genomic-based targets lead a new science of chemical genomics and massive high throughput screening, such mechanism-based screening on the targets presented the limitations: (1) economically unsustainable, (2) difficult to implement, and (3) failed to enhance or improve drug discovery productivity^28^. In order to maximally use known targets and compounds, the approach (selective *single compound* for a *single target*) should be revised. In this respect, chemocentric as well as molecular biology centric need to be considered for screening efficiency. For an extreme example, when the best *single compound* for a *single target* is located in a saddle point (minimax point), the best compound (local maximum) in the target dimension but the worst target (local minimum) in the scaffold dimension, we cannot recognize the situation in current target-based screening (Fig. 7). As the history of screening approaches was taken into consideration in the design of the TR dual screening, we considered target centric and chemo centric dimensions for the bi-directional TR screening. Expectedly, bi-direction of the TR screening clearly showed the best ring system on a target cannot be guaranteed as the best TR pair without the result of inverse target screening of the ring system (see supplementary-file 2). For example, in target dimensions, cyproheptadine was not only the best ligand for RAR β but also the best ligand for fatty acid-binding protein adipocyte (FABP) or liver X receptor α (RXR α) as shown in TR matrix (see Table 1, supplementary-file 2, metrix1). However, ring system dimensions indicated RAR β is the best, FABP is 12th ranked, and RXR α is 17th ranked against cyproheptadine. RAR β-cyproheptadine is the best TR pair in the bi-directional screening (supplementary-file 2, metrix2). Even though we used TR screening for the repurposing of rare ring systems, the TR screening method can apply for inverse target screening of in-house focused library and target for the chosen disease through adjusting dimensions of TR matrix. Finally, how to elicit desirable real compounds from a chosen TR pair only need to be solved by in silico combinatorial screening (generation of virtual library and docking).

### Structure–activity relationship of cyproheptadine

The binding pocket of RAR β (4JYG) is hydrophobic in nature, as most amino acids belong to aliphatic or hydrophobic amino acid residues. In addition to hydrophobic interactions of the tested molecules, its binding affinity to RAR β is further strengthened by H-bond from hydrophilic amino acid residues. Non-covalent interactions with amino acid residues (Table 4) suggest the SAR of cyproheptadine derivatives. In the binding mode, the compound 6b and 6c both make the π–π interactions with Phe279 and hydrogen bond with Arg269 and Ser280 residues respectively. When comparing the binding affinity with compound 6d and 8a, it is revealed that, despite having the critical carboxylic group in the same position with compound 6c, the lower binding affinity of these compounds may be due to lack of π–π interactions to be well-matched with a decreased level of RAR β in Fig. 5C. The nature of binding pocket within 3 Å is Aromatic hydrophobic (Phe192, Trp218, Phe221, Phe279, Phe295), Aliphatic hydrophobic (Leu224, Ala225, Leu259, Leu262, Ile263, Ile266, Leu298, Ile380, Val288, Leu391, Ile403, Met407), Polar, uncharged (Cys228, Ser280) and positively charged (Arg269) amino acid residues. While the hydrophobically enriched binding pocket was complementary to the bound ligand and imparted the binding affinity, the presence of substituents and its orientation contributed to the differences in binding affinity observed. Likewise, the carboxylic acid at the R3 position imparted better binding affinity by hydrogen bonding with Arg269 and Ser280, where compounds containing these groups are stabilized in the binding pocket. Changes in the position of carboxylic acid greatly reduce the binding affinity (i.e. compounds 6a, 6e, and 6f) in Table 2 and Fig. 5C. Moreover, the presence of polar groups like –NO_2,_ or –NH_2_ at the R1 position did not increase the binding affinity (compounds 6d and 8a). Similarly, the low binding affinity has been shown by compound 6f, which contains carboxylic acid at the same position. Thus, it is quite evident from docking studies that, presence of hydrophobic groups increases the binding affinity, which further strengthens by the suitable positioning of hydrophilic groups. Although the common –COOH functionality at R3 position is complementary to binding amino acid residues (i.e. Ser280, Arg269), the presence of other substituents and their juxtaposition in the binding pocket might have caused such difference in experimental activity.

**Table 4:** The binding mode of synthesized N-arylated cyproheptadine derivatives against RAR β target protein (PDB ID: 4JYG).

Compound ID	Docking score	Amino acid residues within 3 Å	Amino acid residues involved in interaction
6a	− 11.506	Phe192, Trp218, Phe221, Leu224, Ala225, Cys228, Arg265, Ile266, Arg269, Phe279, Ser280, Gly294, Phe295, Leu298, Gly384, Val388, Leu391, Arg387, Met399, Ile403, Met407	Phe279 (π–π)
6b	− 15.324	Phe192, Trp218, Phe221, Leu224, Ala225, Cys228, Leu259, Leu262, Ile263, Arg265, Ile266, Arg269, Phe279, Ser280, Gly294, Phe295, Leu298, Ile380, Val388, Leu391, Met399, Ile403	Arg269 and Ser280 (H-bond), Phe279 (π–π)
6c	− 15.568	Phe192, Leu259, ILeu262, Ile263, Arg265, le266, Arg269, Trp218, Phe221, Leu224, Ala225, Cys228, Phe279, Ser280, Phe295, Leu298, Ile380, Val388, Leu391, Ile403, Met407	Arg269 and Ser280 (H-bond), Phe279 (π–π)
6d	− 12.22	Phe192, Trp218, Phe221, Leu224, Ala225, Cys228, Leu259, Leu262, Ile263, Arg265, Ile266, Arg269, Phe279, Ser280, Phe295, Leu298, Ile380, Val388, Leu391, Met399, Ile403	Arg269 and Ser280 (H-bond)
6e	− 11.569	Phe192, Trp218, Phe221, Leu224, Ala225, Cys228, Leu259, Leu262, Ile263, Arg265, le266, Arg269, Phe279, Ser280, Gly294, Phe295, Leu298, Ile380, Val388, Leu391, Ile403, Met407	Phe279 (π–π)
6f.	− 8.404	Phe192, Trp218, Phe221, Leu224, Ala225, Cys228, Leu259, Leu262, Ile263, Arg265, ile266, Arg269, Phe279, Phe295, Leu298, Val388, Leu391, Ile403	–
8a	− 12.994	Phe192, Trp218, Phe221, Leu224, Ala225, Cys228, Leu259, Leu262, Ile263, Arg265, Ile266, Arg269, Phe279, Ser280, Gly294, Phe295, Leu298, Ile380, Gly384, Val388, Leu391, Met399, Ile403	Arg269 and Ser280 (H-bond)
8b	− 14.352	Phe192, Trp218, Phe221, Leu224, Ala225, Cys228, Leu259, Leu262, Ile263, Arg265, Ile266, Arg269, Phe279, Ser280, Phe295, Leu298, Val388, Leu391, Ile403	Arg269 and Ser280 (H-bond), Phe279 (π–π)

## Experiments and methods

### Collection and preparation of the dataset

The compound data containing ChEMBL ID and canonical smiles as 1,583,897 single entries were downloaded from the ChEMBL version 21. The structural information-carrying SMILES was converted and molecular properties were calculated by using the CDK toolkit^29^. All biological activity data were downloaded additionally and assigned to compound data. Similarly, the protein data containing protein number as 6930 single entries were downloaded from the ChEMBL version 21. Furthermore, from PDB bank, 219,393 ligands without hydrogen in 3D structures of deposited proteins were downloaded from the protein data bank. As a criterion for protein filtration, protein accession id was assigned to PDB and further labeled with pharmaceutical development step information, which was downloaded from the Therapeutic Target Database^10^. The above following steps ensured the final selection of research/clinical trial targets.

### Preparation of ligands

The ligands were prepared using the LigPrep module of the Schrodinger suite^30^. The LigPrep generates multiple states tautomers, stereoisomers, desaltation, and ionization at a pH range (7 ± 2) using Epik method^31^. The default settings were employed to enumerate 3D conformers with OPLS_2005 force field^32^.

### Preparation of proteins

The protein structures were prepared using the Protein Preparation Wizard^33^. Water molecules were deleted, bond orders were assigned, and hydrogen atom was added. Hydrogen bond was assigned using PROPKA (pH 7.0) and finally, structures were refined through restrained minimization until converges within 0.30 Å RMSD for heavy atoms.

### Target (T) and ring system (R) dual screening

In the adjusted scale of both targets and ring systems, a bi-directional TR screening (**r**ing system based **t**arget screening, **t**arget system based **r**ing screening) was conducted. As a preliminary screening, bi-directional docking was neither facile nor productive due to the size of the system (M: the number of PDBs on 97 targets = 3424 PDBs, N: the number of conformation of 71 ring systems = one conformer). Therefore, shape-based calculation of M × N matrix was expected for high speedy performance. At that time, the chemo centric assumption can permit the converted information from PDB structures of 97 targets into ligand conformers of the PDBs. The initial shape-based similarity helps to reduce the large pool of PDBs and feasible for secondary bi-directional docking.

### Shape-based similarity calculation

The Shape-based similarity calculation performed with OpenEye’s ROCS software^34^. ROCS is a popular tool because of its comparison efficiency and rapid speed. Before running ROCS calculation, between rings and PDB’s ligand structure, the ligand structures in PDB was filtered for druggability by assigning TTD database information to ligand entries and excluding entries label with the successful target. Additionally, inorganic compounds and compounds with molecular weight beyond the range of 250–800 were excluded. Similarly, the virtual libraries were filtered based on predicted molecular properties (HERG score < − 5, oral > 80%, Rule of five < 2, rule of three < 1, and protonated amide compound was deleted). The ROCS was designed to perform the Shape-based similarity search with pharmacophore features and it was conducted by comparing the shape of conformers of X-ray ligand as a reference set and ring structure as well as virtual libraries as query molecules^35,36^. Shape similarity calculation based on the Tanimoto combo score (the summation of two Tanimoto coefficients from molecular shape and color features) and during calculation Tanimoto cut off score was set to − 0.1 to also include low score result. The maximum result number was set to 500 based on the high order of the Tanimoto combo score. However, we observed narrow distribution and deviation of calculated Tanimoto combo score. So further ranking of ROCS result, we created a new score (fused score). It is well-known that multiplication is more sensitive to the variance between each values (less or more than score 1) rather than summation^37^. In other words, multiplication can discriminate distinct values with others among values of raw data. Thus, multiplication of similarity scores was used in this study. In detail, for each similarity scores calculated for each X-ray ligands, the basic statistical values, such as the maximum, minimum and mean values were calculated. From shape similarity score matrix, the Ref Tversky Combo Max, Tanimoto Combo Mean, Ref Tversky Combo Mean, Tanimoto Combo Max, Ref Tversky Combo Min, Color Tanimoto Min, count scores were multiplied to each other, resulting in new scores (fused score) and sorted accordingly^37,38^.

$$ {\text{Fused score}}\, = \,{\text{RefTverskyCombo}}\_{\text{Max}}\, \times \,{\text{TanimotoCombo}}\_{\text{Mean}}\, \times \,{\text{RefTverskyCombo}}\_{\text{Mean}}\, \times \,{\text{TanimotoCombo}}\_{\text{Max}}\, \times \,{\text{RefTverskyCombo}}\_{\text{Min}}\, \times \,{\text{ColorTanimoto}}\_{\text{Min}}\, \times \,{\text{count}} $$

### Molecular docking simulations

The SP (standard precision) and XP (Xtra precision) protocol of the Glide^39^ module were utilized to dock the Rings and ligand libraries to the protein receptor. The receptor grid was defined based on bound X-ray ligand with inner boxes lengths of 10 Å × 10 Å × 10 Å. The scaling factor of van der Waals radius was set to 1.0 and partial charge cutoff was set to 0.25. The centroid of the outer box was based on X-ray ligand and ligand libraries were docked flexibly under the default settings. The post-docking minimization was performed with the threshold for rejecting minimized pose and the number of pose per ligand to included was set to 0.50 kcal/mol and 10, respectively.

### Virtual library design and prediction of ADMET properties

The virtual compound libraries were generated using combiglide^40^. A reagent database (virtual reagents) limited to aryl or vinyl bromide and commercially available library in ZINC database was generated from Alfa-aesar database with multiple state tautomers, stereoisomers, ionization at a pH range (7 ± 2). The generated virtual libraries were untangled and minimized. The prepared ligand libraries were further predicted for their octanol/water partition, log BB, overall CNS activity, Caco-2, and MDCK cell permeability, logKhsa for human serum albumin binding and log IC_50_ for HERG K+ channel blockage as Absorption, Distribution, Metabolism, Excretion and Toxicity properties using QikProp module of Schrodinger suite^41^.

### Nuclear receptor reporter assay

The human RAR β reporter assay was performed according to the kit protocol in a 3 × 32 assays in a 96 well plate format (Indigo No: #IB02101-32, PA, 16801, USA). As a kind of reporter gene assay, Luciferase was inserted as a reporter gene into a regulatory sequence of RAR β. RAR β reporter cells were seeded in a 96 well plate that has been provided with the kit and treated with various concentrations of negative and positive control to measure transcriptional activity. Treated cell plate was incubated into a 37 °C, humidified with 5% CO_2_ incubator for about 22–24 h. On the next day, the supernatant was removed and the Luciferase detection reagent was added. The light emission from each assay well has been quantified using luminescence system.

## Conclusion

In this study, we have developed bi-directional TR dual screening to be complementary to drawback of general drug repurposing. Under the method, the optimal target-ring system pair of RAR β and cyproheptadine could be identified with falling in the saddle point between target centric dimensions and chemocentric dimensions. Compounds **6c** showed concentration dependent agonism on RAR β with micromolar level potency. In the future, synergic effect between known targets of the ring system and RAR β can be further investigated in a modified TR matrix from a chosen axis such as SETD7-ERα-RAR β.

## Supplementary information

Supplementary Information.

Supplementary Information.

## Data Availability

Knime workflow and refined data will be available in GitHub. https://github.com/college-of-pharmacy-gachon-university/TRScreening.
