# Supplementary material for: Repurposing of FDA approved ring systems through bi-directional target-ring system dual screening
Source: Sci Rep. 2020 Dec 3;10:21133. doi: 10.1038/s41598-020-78077-9 (PMC7713353; doi:10.1038/s41598-020-78077-9)
Supplement: Supplementary file 1 — Supplementary Information. [file 41598_2020_78077_MOESM1_ESM.docx]

**Supplementary Material**

**Repurposing of FDA Approved Ring Systems Through Bi-directional Target-Ring System Dual Screening**

Surendra Kumar ^ǂ^, Cheongyun Jang^ǂ^, Lalita Subedi, Sun Yeou Kim, Mi-hyun Kim^*^

Gachon Institute of Pharmaceutical Science & Department of Pharmacy, College of Pharmacy, Gachon University, 191 Hambakmoeiro, Yeonsu-gu, Incheon, Republic of Korea

^ǂ^ The authors are co-first authors

^*^Author for correspondence

E-mail: [kmh0515@gachon.ac.kr](mailto:kmh0515@gachon.ac.kr)

**Contents:**

1. **Table S1: Ring structures from Small Molecule Drugs Listed in the FDA Orange Book with frequencies 2 or less.**
2. **Table S2: 3D similarity based shape screen result (Best Target)**
3. **Table S3: 3D similarity based shape screen result (Best Ring)**
4. **Table S4: Docking based secondary TR screening result: Best ring –target pairs**
5. **Materials and Methods**
   1. **Synthetic Chemistry**
      1. **General procedure of chemistry**
6. **Results**
   1. **Synthesis of cyproheptadine derivatives and reagent library**
   2. **Procedure for synthesis of starting compound (4)**
   3. **General Procedure for the Synthesis of 5 (a-g)**
   4. **General Procedure for the Synthesis of 6 (a-g)**
   5. **General Procedure for the Synthesis of 7(a-b)**
   6. **General Procedure for the Synthesis of 8 (a-b)**
   7. **Synthesis of reagent library and target compound**
   8. **General Procedure for the Synthesis of 9(a-c)**
   9. **General Procedure for the Synthesis of 10(a-d)**

**Table S1: Ring structures from Small Molecule Drugs Listed in the FDA Orange Book with frequencies 2 or less.**

| **S. No.** | **Ring-Id** | **Structure** | **FC** | **HA** | **HD** | **MW** | **VABC** |
| --- | --- | --- | --- | --- | --- | --- | --- |
| **1** | **117-124_7** | 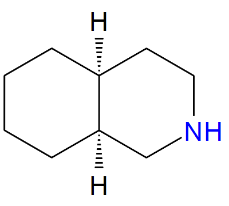 | 694.01 | 1 | 1 | 139.14 | 150.50 |
| **2** | **242-250_0** | 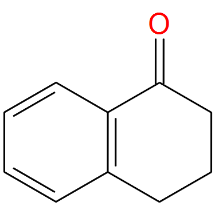 | 374.01 | 1 | 0 | 146.07 | 144.15 |
| **3** | **125-131_1** | 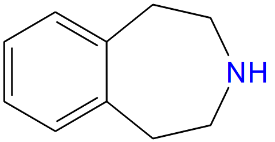 | 515.01 | 1 | 1 | 147.10 | 148.99 |
| **4** | **242-250_3** | 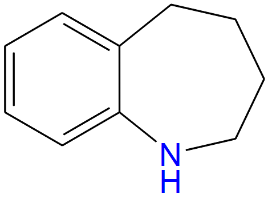 | 515.01 | 1 | 1 | 147.10 | 148.99 |
| **5** | **125-131_3** | 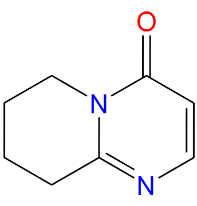 | 374.03 | 1 | 0 | 150.08 | 145.09 |
| **6** | **251-256_0** | 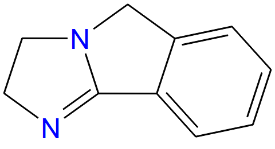 | 444.02 | 2 | 0 | 158.08 | 144.99 |
| **7** | **251-256_1** | 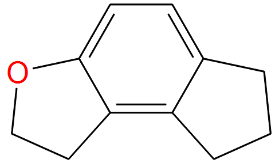 | 544.01 | 0 | 0 | 160.09 | 151.72 |
| **8** | **251-256_5** | 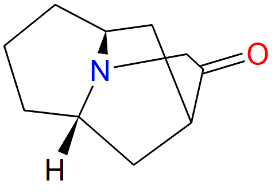 | 709.02 | 2 | 0 | 165.12 | 161.60 |
| **9** | **125-131_5** | 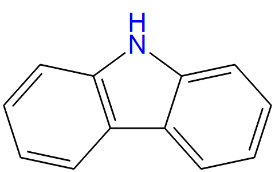 | 420.01 | 0 | 1 | 167.07 | 141.52 |
| **10** | **257-262_2** | 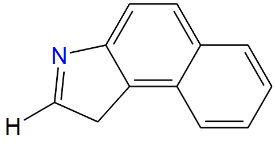 | 420.01 | 1 | 0 | 167.07 | 152.42 |
| **11** | **257-262_0** | 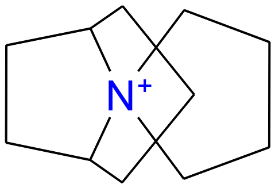 | 1093.01 | 1 | 1 | 167.17 | 168.78 |
| **12** | **257-262_4** | 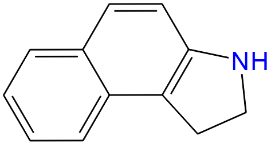 | 520.01 | 1 | 1 | 169.09 | 155.05 |
| **13** | **257-262_5** | 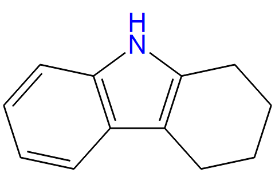 | 628.01 | 0 | 1 | 171.10 | 157.69 |
| **14** | **263-268_0** | 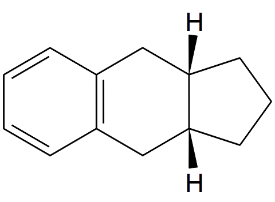 | 805.00 | 0 | 0 | 172.13 | 177.53 |
| **15** | **263-268_3** | 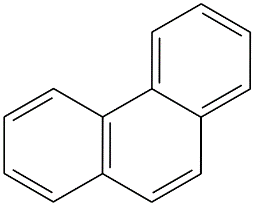 | 494.00 | 0 | 0 | 178.08 | 162.48 |
| **16** | **263-268_4** | 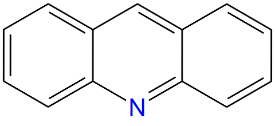 | 443.01 | 1 | 0 | 179.07 | 156.18 |
| **17** | **269-273_0** | 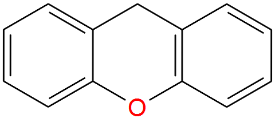 | 494.01 | 0 | 0 | 182.07 | 167.51 |
| **18** | **269-273_1** | 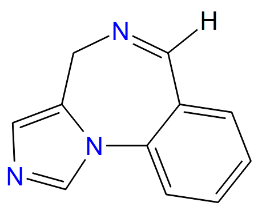 | 443.03 | 2 | 0 | 183.08 | 157.11 |
| **19** | **269-273_2** | 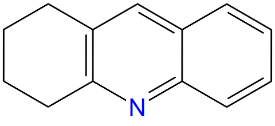 | 659.01 | 1 | 0 | 183.10 | 172.35 |
| **20** | **269-273_4** | 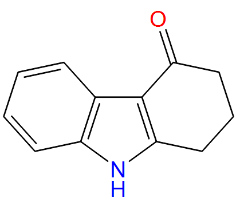 | 547.02 | 1 | 1 | 185.08 | 163.84 |
| **21** | **274-279_2** | 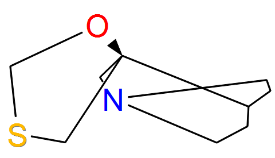 | 709.03 | 2 | 0 | 185.09 | 165.45 |
| **22** | **132-137_0** | 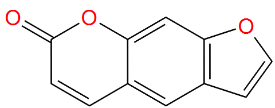 | 302.03 | 0 | 0 | 186.03 | 150.49 |
| **23** | **274-279_4** | 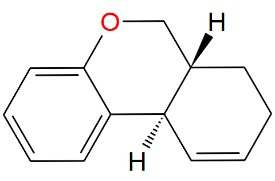 | 718.01 | 0 | 0 | 186.10 | 183.68 |
| **24** | **274-279_5** | 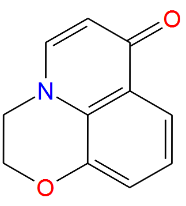 | 443.03 | 0 | 0 | 187.06 | 166.24 |
| **25** | **132-137_2** | 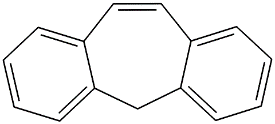 | 631.00 | 0 | 0 | 192.09 | 190.67 |
| **26** | **132-137_1** | 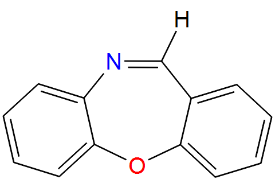 | 466.02 | 1 | 0 | 195.07 | 175.87 |
| **27** | **286-291_1** | 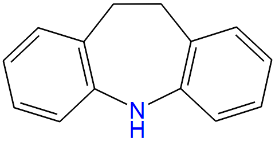 | 690.01 | 1 | 1 | 195.10 | 187.01 |
| **28** | **132-137_4** | 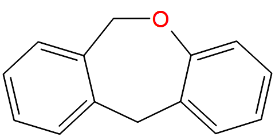 | 631.01 | 0 | 0 | 196.09 | 184.80 |
| **29** | **286-291_2** | 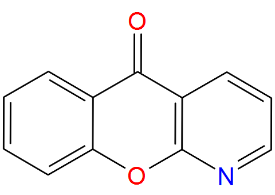 | 366.03 | 1 | 0 | 197.05 | 167.36 |
| **30** | **286-291_5** | 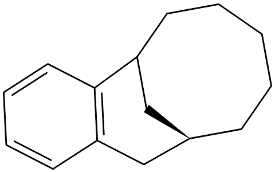 | 1159.00 | 0 | 0 | 200.16 | 212.12 |
| **31** | **292-296_0** | 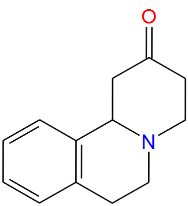 | 814.02 | 2 | 0 | 201.12 | 194.68 |
| **32** | **292-296_2** | 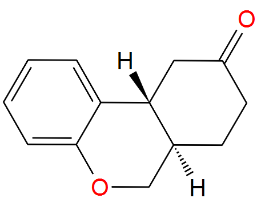 | 751.02 | 1 | 0 | 202.10 | 192.47 |
| **33** | **292-296_4** | 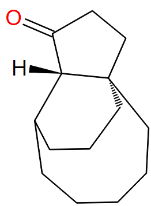 | 1311.01 | 1 | 0 | 206.17 | 219.78 |
| **34** | **297-302_0** | 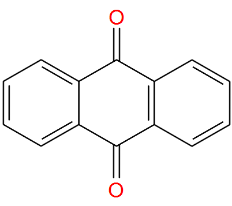 | 436.02 | 2 | 0 | 208.05 | 188.32 |
| **35** | **297-302_4** | 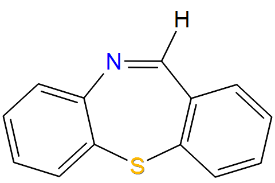 | 466.02 | 1 | 0 | 211.05 | 185.59 |
| **36** | **303-308_1** | 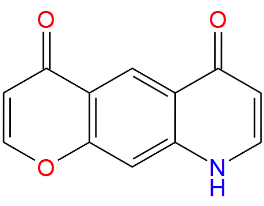 | 385.04 | 0 | 1 | 213.04 | 187.05 |
| **37** | **303-308_2** | 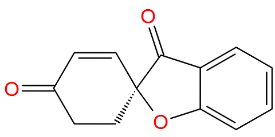 | 544.03 | 2 | 0 | 214.06 | 195.99 |
| **38** | **303-308_3** | 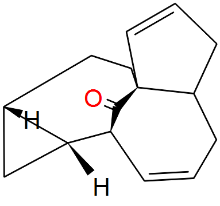 | 1129.01 | 1 | 0 | 214.14 | 219.45 |
| **39** | **303-308_4** | 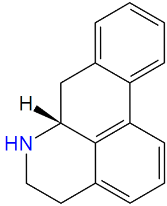 | 953.01 | 1 | 1 | 221.12 | 209.24 |
| **40** | **303-308_5** | 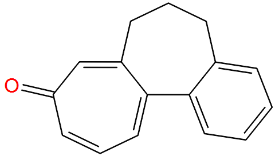 | 817.01 | 0 | 0 | 222.10 | 227.66 |
| **41** | **309-314_0** | 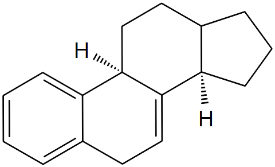 | 1328.00 | 0 | 0 | 224.16 | 231.72 |
| **42** | **309-314_2** | 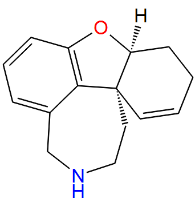 | 1097.02 | 1 | 1 | 227.13 | 216.91 |
| **43** | **309-314_3** | 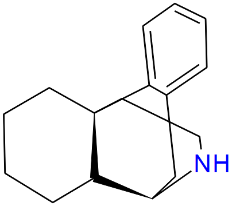 | 1409.01 | 1 | 1 | 227.17 | 228.05 |
| **44** | **309-314_4** | 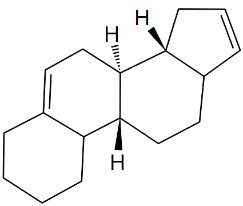 | 1664.00 | 0 | 0 | 228.19 | 247.89 |
| **45** | **132-137_5** | 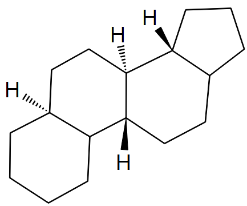 | 2032.00 | 0 | 0 | 232.22 | 253.16 |
| **46** | **315-318_1** | 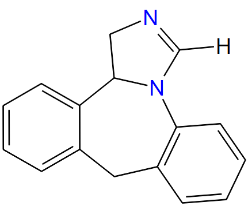 | 919.02 | 2 | 0 | 234.12 | 217.60 |
| **47** | **315-318_2** | 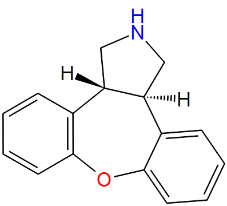 | 990.02 | 1 | 1 | 237.12 | 218.03 |
| **48** | **315-318_3** | 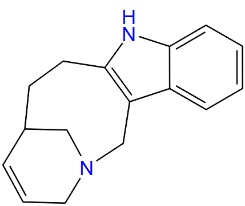 | 1215.02 | 1 | 1 | 238.15 | 222.88 |
| **49** | **319-324_0** | 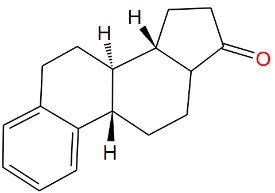 | 1375.01 | 1 | 0 | 240.15 | 240.51 |
| **50** | **138-143_0** | 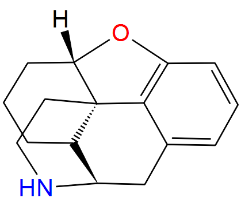 | 1375.02 | 1 | 1 | 241.15 | 224.49 |
| **51** | **319-324_1** | 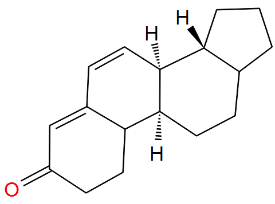 | 1543.01 | 1 | 0 | 242.17 | 254.04 |
| **52** | **319-324_2** | 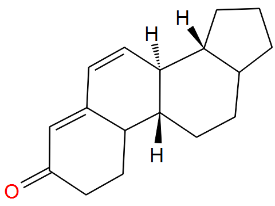 | 1543.01 | 1 | 0 | 242.17 | 254.04 |
| **53** | **138-143_1** | 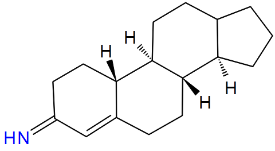 | 1810.01 | 1 | 1 | 243.20 | 258.89 |
| **54** | **319-324_3** | 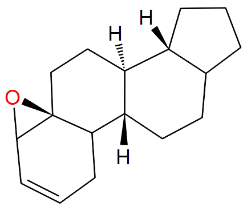 | 1810.01 | 1 | 0 | 244.18 | 246.96 |
| **55** | **319-324_4** | 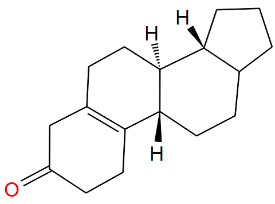 | 1719.01 | 1 | 0 | 244.18 | 256.68 |
| **56** | **319-324_5** | 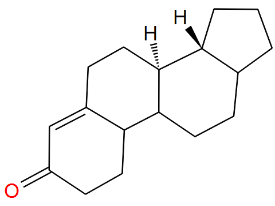 | 1719.01 | 1 | 0 | 244.18 | 256.68 |
| **57** | **138-143_3** | 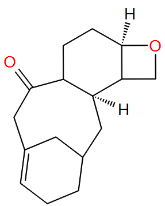 | 1543.02 | 2 | 0 | 246.16 | 248.17 |
| **58** | **325_328_1** | 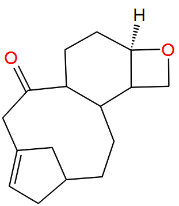 | 1543.02 | 2 | 0 | 246.16 | 248.17 |
| **59** | **138-143_4** | 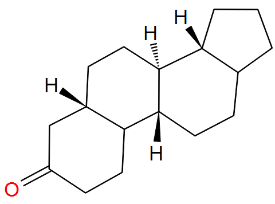 | 1903.01 | 1 | 0 | 246.20 | 259.32 |
| **60** | **325-328_0** | 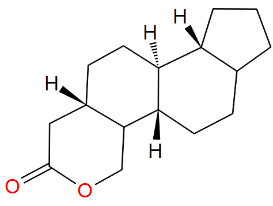 | 1719.02 | 2 | 0 | 248.18 | 250.81 |
| **61** | **144-147_0** | 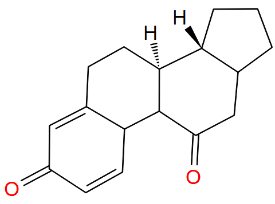 | 1422.02 | 2 | 0 | 256.15 | 260.20 |
| **62** | **329-333_2** | 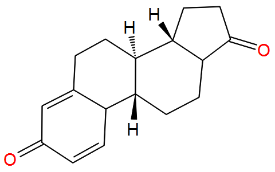 | 1422.02 | 2 | 0 | 256.15 | 260.20 |
| **63** | **329-333_3** | 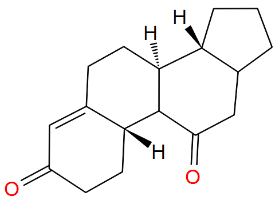 | 1594.02 | 2 | 0 | 258.16 | 262.83 |
| **64** | **144-147_1** | 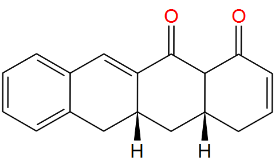 | 1141.02 | 2 | 0 | 264.12 | 258.68 |
| **65** | **334-338_1** | 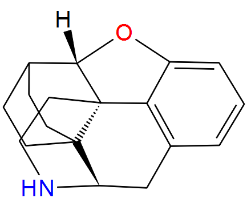 | 1736.02 | 1 | 1 | 267.16 | 246.72 |
| **66** | **334-338_3** | 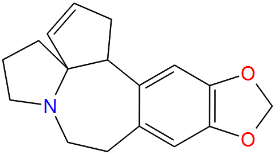 | 1469.03 | 1 | 0 | 269.14 | 247.94 |
| **67** | **334-338_2** | 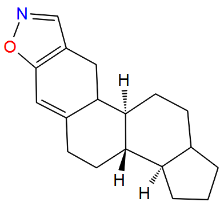 | 1829.02 | 0 | 0 | 269.18 | 259.08 |
| **68** | **334-338_4** | 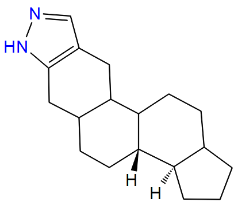 | 2120.02 | 1 | 1 | 270.21 | 263.92 |
| **69** | **339-342_1** | 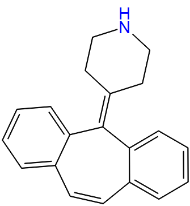 | 1429.01 | 1 | 1 | 273.15 | 273.16 |
| **70** | **144-147_2** | 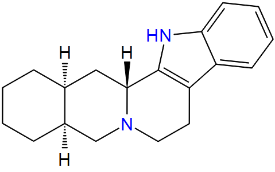 | 1981.02 | 1 | 1 | 280.19 | 265.05 |
| **71** | **347-349_2** | 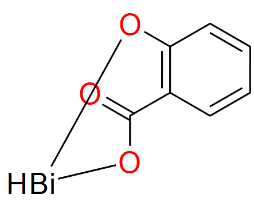 | 179.04 | 2 | 0 | 346.00 | NaN |

**Abbreviations:** FC: Fragment Complexity; HA: Hydrogen Bond Acceptor; HD: Hydrogen Bond Donor; MW: Molecular Weight; VABC: Atomic and Bond Contributions of van der Waals volume

**Table S2: 3D similarity based shape screen result: Best target for query ring systems**

| **Database** | **Shape_Sim** | **Query** | **PDB-ID** | **UniprotID** |
| --- | --- | --- | --- | --- |
| 3ITU_IBM_A_999 | 0.6807 | 286-291_2 | 3ITU | O00408 |
| 3FF6_RCP_A_1 | 0.4487 | 286-291_2 | 3FF6 | O00763 |
| 5BQG_JZR_A_203 | 0.6253 | 319-324_3 | 5BQG | O14684 |
| 3O34_BTN_B_1 | 0.6278 | 329-333_2 | 3O34 | O15164 |
| 2EVA_ADN_A_498 | 0.6019 | 286-291_2 | 2EVA | O43318 |
| 5J79_6GE_A_401 | 0.5290 | 303-308_1 | 5J79 | O43353 |
| 4QB3_30M_A_201 | 0.6847 | 269-273_4 | 4QB3 | O60885 |
| 4Z7H_4L5_A_1001 | 0.4860 | 303-308_1 | 4Z7H | O75460 |
| 4X1G_3WF_A_501 | 0.6848 | 309-314_0 | 4X1G | O75469 |
| 4I3K_1BX_A_505 | 0.7144 | 303-308_5 | 4I3K | O75874 |
| 4MD6_24E_A_902 | 0.7507 | 286-291_2 | 4MD6 | O76074 |
| 3JSI_WTC_A_903 | 0.6867 | 286-291_2 | 3JSI | O76083 |
| 1XAN_HXP_A_1024 | 0.7886 | 269-273_0 | 1XAN | P00390 |
| 1QFW_NAG_A_93 | 0.6083 | 251-256_5 | 1QFW | P01215 |
| 2WQA_OLA_E_1177 | 0.5694 | 132-137_5 | 2WQA | P02753 |
| 4DER_AGI_A_201 | 0.7038 | 286-291_2 | 4DER | P02766 |
| 3UU7_2OH_A_1 | 0.7980 | 132-137_2 | 3UU7 | P03372 |
| 4P6X_HCY_A_900 | 0.6441 | 329-333_2 | 4P6X | P04150 |
| 1ZNQ_NAD_O_1001 | 0.2547 | 286-291_2 | 1ZNQ | P04406 |
| 2VUK_P83_B_1291 | 0.7023 | 125-131_5 | 2VUK | P04637 |
| 4FR8_TNG_A_601 | 0.6484 | 347-349_2 | 4FR8 | P05091 |
| 4NKZ_LZZ_A_601 | 0.7201 | 334-338_2 | 4NKZ | P05093 |
| 1P53_NAG_A_1240 | 0.6321 | 251-256_5 | 1P53 | P05362 |
| 3HG4_7JZ_A_801 | 0.8080 | 292-296_4 | 3HG4 | P06280 |
| 4EEH_HH6_A_301 | 0.7114 | 263-268_4 | 4EEH | P07900 |
| 5A1P_STR_A_1600 | 0.6652 | 334-338_2 | 5A1P | P08684 |
| 3KMO_EAA_A_214 | 0.5688 | 329-333_3 | 3KMO | P09211 |
| 3ZQT_30Z_A_1920 | 0.7758 | 309-314_0 | 3ZQT | P10275 |
| 4JYG_1NY_A_501 | 0.5637 | 138-143_0 | 4JYG | P10826 |
| 2PIN_LEG_A_501 | 0.6959 | 309-314_0 | 2PIN | P10828 |
| 5EX5_7DD_A_503 | 0.4030 | 132-137_0 | 5EX5 | P11021 |
| 1HNN_SKF_A_3001 | 0.7146 | 347-349_2 | 1HNN | P11086 |
| 1SC7_M38_C_990 | 0.5103 | 269-273_4 | 1SC7 | P11387 |
| 1Z11_8MO_A_501 | 0.7796 | 132-137_0 | 1Z11 | P11509 |
| 1A27_EST_A_350 | 0.7968 | 309-314_0 | 1A27 | P14061 |
| 5J7Q_6H2_A_201 | 0.8147 | 263-268_4 | 5J7Q | P14174 |
| 3U8D_U8D_A_201 | 0.5306 | 132-137_0 | 3U8D | P14555 |
| 4JPG_1OX_B_602 | 0.6082 | 286-291_2 | 4JPG | P14618 |
| 4QFD_31R_A_403 | 0.6925 | 132-137_0 | 4QFD | P14920 |
| 2HNX_PLM_A_135 | 0.6792 | 132-137_5 | 2HNX | P15090 |
| 1E1Z_NDG_P_601 | 0.6221 | 251-256_5 | 1E1Z | P15289 |
| 1H66_RH1_A_1274 | 0.5579 | 292-296_4 | 1H66 | P15559 |
| 4WF6_407_A_802 | 0.6182 | 334-338_3 | 4WF6 | P15917 |
| 1ZX1_CB1_A_233 | 0.7201 | 242-250_0 | 1ZX1 | P16083 |
| 3NSQ_CHZ_B_1 | 0.9919 | 297-302_0 | 3NSQ | P19793 |
| 3QU8_3QU_A_501 | 0.6704 | 286-291_5 | 3QU8 | P20813 |
| 4REF_3N0_A_302 | 0.6885 | 242-250_0 | 4REF | P22736 |
| 1MX9_NLX_A_1 | 0.7370 | 138-143_0 | 1MX9 | P23141 |
| 4A9I_P9I_A_1185 | 0.5053 | 263-268_4 | 4A9I | P25440 |
| 3SA0_NRA_A_361 | 0.8407 | 286-291_2 | 3SA0 | P28482 |
| 3BYZ_H11_A_601 | 0.6488 | 274-279_2 | 3BYZ | P28845 |
| 4G2F_C07_A_1001 | 0.5924 | 132-137_0 | 4G2F | P29320 |
| 4D1P_H4B_A_600 | 0.8205 | 125-131_3 | 4D1P | P29474 |
| 3ARN_MSJ_A_165 | 0.4047 | 325-328_1 | 3ARN | P33316 |
| 5AI6_4XH_A_1551 | 0.7983 | 269-273_2 | 5AI6 | P34913 |
| 5EQP_5R9_A_501 | 0.6619 | 263-268_4 | 5EQP | P35790 |
| 5AFK_L0B_A_1205 | 0.6247 | 303-308_3 | 5AFK | P36544 |
| 4X2F_3WJ_A_601 | 0.6975 | 286-291_2 | 4X2F | P36897 |
| 5E8Y_STU_A_601 | 0.4606 | 334-338_1 | 5E8Y | P37173 |
| 4JAZ_STL_A_501 | 0.6956 | 132-137_2 | 4JAZ | P37231 |
| 2WJW_NAG_A_1399 | 0.5843 | 251-256_5 | 2WJW | P42262 |
| 4W9N_TCL_B_1905 | 0.5321 | 132-137_1 | 4W9N | P49327 |
| 1Q3W_ATU_A_502 | 0.7043 | 315-318_1 | 1Q3W | P49841 |
| 3NBW_TR4_A_301 | 0.6054 | 274-279_2 | 3NBW | P50053 |
| 1R5H_AO2_A_501 | 0.5672 | 292-296_4 | 1R5H | P50579 |
| 5G44_YTZ_A_1512 | 0.5669 | 347-349_2 | 5G44 | P51449 |
| 3K5E_K5E_A_369 | 0.7687 | 263-268_0 | 3K5E | P52732 |
| 2OWB_626_A_500 | 0.4424 | 132-137_0 | 2OWB | P53350 |
| 4FLI_Y16_A_503 | 0.5936 | 144-147_1 | 4FLI | P53582 |
| 1PMU_9HP_A_501 | 0.6490 | 132-137_0 | 1PMU | P53779 |
| 1PQ9_44B_A_1501 | 0.6358 | 286-291_5 | 1PQ9 | P55055 |
| 5FTJ_OJA_A_1001 | 0.4425 | 315-318_3 | 5FTJ | P55072 |
| 5E3D_5JL_A_801 | 0.7079 | 269-273_2 | 5E3D | P55201 |
| 3STD_MQ0_A_503 | 0.6061 | 286-291_2 | 3STD | P56221 |
| 1HAC_NDD_B_400 | 0.6726 | 257-262_2 | 1HAC | P68871 |
| 4OXY_1TN_A_302 | 0.7218 | 269-273_0 | 4OXY | P9WGR1 |
| 3KVL_DET_A_400 | 0.6925 | 132-137_5 | 3KVL | Q02127 |
| 3PEQ_JZR_A_478 | 0.6163 | 319-324_3 | 3PEQ | Q03181 |
| 4DYM_IYZ_A_503 | 0.5564 | 286-291_2 | 4DYM | Q04771 |
| 1RO6_ROL_A_601 | 0.6082 | 334-338_3 | 1RO6 | Q07343 |
| 1U4D_DBQ_A_398 | 0.6665 | 292-296_2 | 1U4D | Q07912 |
| 5JAR_6HV_A_504 | 0.5760 | 286-291_2 | 5JAR | Q13093 |
| 5AVI_4KM_A_501 | 0.4304 | 347-349_2 | 5AVI | Q13133 |
| 3I6C_GIA_A_301 | 0.6724 | 286-291_5 | 3I6C | Q13526 |
| 5A3X_QIV_A_1482 | 0.7423 | 269-273_2 | 5A3X | Q13627 |
| 3H3C_P1E_A_999 | 0.4107 | 334-338_2 | 3H3C | Q14289 |
| 4MP7_PFT_A_501 | 0.6090 | 274-279_4 | 4MP7 | Q15119 |
| 4EH7_0OO_A_402 | 0.7340 | 269-273_0 | 4EH7 | Q16539 |
| 2C11_NAG_A_1730 | 0.6084 | 251-256_5 | 2C11 | Q16853 |
| 4D4L_BKS_A_1449 | 0.6362 | 286-291_2 | 4D4L | Q16875 |
| 5IID_6BK_A_801 | 0.6914 | 286-291_2 | 5IID | Q86U86 |
| 4NYV_15E_A_1201 | 0.6658 | 251-256_0 | 4NYV | Q92793 |
| 4BIB_IEO_A_1940 | 0.6046 | 274-279_5 | 4BIB | Q99683 |
| 5F2P_5TY_A_201 | 0.6418 | 286-291_2 | 5F2P | Q9H8M2 |
| 1RFF_SPM_A_700 | 0.0000 | 117-124_7 | 1RFF | Q9NUW8 |
| 4G34_924_A_1101 | 0.4906 | 132-137_0 | 4G34 | Q9NZJ5 |
| 5CUA_54U_A_2002 | 0.6710 | 263-268_0 | 5CUA | Q9UIF8 |

**Note:** Database represent: PdbID_LigandID_Chain_NumberOfResidues

**Table S3: 3D similarity based shape screen result: Best ring for query targets**

| **Database** | **Shape_Sim** | **Ring-ID** | **Query** | **UniprotID** |
| --- | --- | --- | --- | --- |
| 3K5E_K5E_A_369 | 0.6823 | 117-124_7 | 3K5E | P52732 |
| 3I6C_GIA_A_301 | 0.6576 | 125-131_1 | 3I6C | Q13526 |
| 4D1P_H4B_A_600 | 0.8205 | 125-131_3 | 4D1P | P29474 |
| 2VUK_P83_B_1291 | 0.7023 | 125-131_5 | 2VUK | P04637 |
| 1Z11_8MO_A_501 | 0.7796 | 132-137_0 | 1Z11 | P11509 |
| 1XAN_HXP_A_1024 | 0.6792 | 132-137_1 | 1XAN | P00390 |
| 3UU7_2OH_A_1 | 0.7980 | 132-137_2 | 3UU7 | P03372 |
| 3EAH_327_A_864 | 0.7103 | 132-137_4 | 3EAH | P29474 |
| 4MGA_27L_A_601 | 0.7625 | 132-137_5 | 4MGA | P03372 |
| 1MX9_NLX_A_1 | 0.7370 | 138-143_0 | 1MX9 | P23141 |
| 2AMB_17H_A_1001 | 0.7106 | 138-143_1 | 2AMB | P10275 |
| 2X7D_EGB_A_1369 | 0.6801 | 138-143_3 | 2X7D | P52732 |
| 3KM0_AOM_C_1 | 0.6834 | 138-143_4 | 3KM0 | P14061 |
| 2AMA_DHT_A_1001 | 0.6884 | 144-147_0 | 2AMA | P10275 |
| 1EQU_EQI_A_329 | 0.6848 | 144-147_1 | 1EQU | P14061 |
| 5DX3_EST_A_601 | 0.6653 | 144-147_2 | 5DX3 | P03372 |
| 4OGH_HFT_A_1001 | 0.7609 | 242-250_0 | 4OGH | P10275 |
| 3I6C_GIA_A_301 | 0.6580 | 242-250_3 | 3I6C | Q13526 |
| 3HG4_7JZ_A_801 | 0.7072 | 251-256_0 | 3HG4 | P06280 |
| 3EAH_327_A_864 | 0.7320 | 251-256_1 | 3EAH | P29474 |
| 3WK4_S0A_A_603 | 0.7070 | 251-256_5 | 3WK4 | P34913 |
| 1M9R_INE_A_906 | 0.7622 | 257-262_2 | 1M9R | P29474 |
| 1M9R_INE_A_906 | 0.7279 | 257-262_4 | 1M9R | P29474 |
| 1M9R_INE_A_906 | 0.7095 | 257-262_5 | 1M9R | P29474 |
| 3K5E_K5E_A_369 | 0.7687 | 263-268_0 | 3K5E | P52732 |
| 1GWQ_ZTW_A_600 | 0.6824 | 263-268_3 | 1GWQ | P03372 |
| 3SA0_NRA_A_361 | 0.8153 | 263-268_4 | 3SA0 | P28482 |
| 1XAN_HXP_A_1024 | 0.7886 | 269-273_0 | 1XAN | P00390 |
| 2CCS_4BH_A_1224 | 0.6371 | 269-273_1 | 2CCS | P07900 |
| 5AI6_4XH_A_1551 | 0.7983 | 269-273_2 | 5AI6 | P34913 |
| 4QB3_30M_A_201 | 0.6847 | 269-273_4 | 4QB3 | O60885 |
| 3WK6_S0B_A_603 | 0.7085 | 274-279_2 | 3WK6 | P34913 |
| 3EAH_327_A_864 | 0.7762 | 274-279_4 | 3EAH | P29474 |
| 1X7R_GEN_A_201 | 0.6506 | 274-279_5 | 1X7R | P03372 |
| 4PP6_STL_A_601 | 0.5884 | 286-291_1 | 4PP6 | P03372 |
| 3SA0_NRA_A_361 | 0.8407 | 286-291_2 | 3SA0 | P28482 |
| 3K5E_K5E_A_369 | 0.7646 | 286-291_5 | 3K5E | P52732 |
| 2G70_HNT_A_5001 | 0.6427 | 292-296_0 | 2G70 | P11086 |
| 2POG_WST_A_201 | 0.7331 | 292-296_2 | 2POG | P03372 |
| 3HG4_7JZ_A_801 | 0.8080 | 292-296_4 | 3HG4 | P06280 |
| 3NSQ_CHZ_B_1 | 0.9919 | 297-302_0 | 3NSQ | P19793 |
| 3EAH_327_A_864 | 0.7154 | 297-302_4 | 3EAH | P29474 |
| 1DVU_DBF_B_125 | 0.6556 | 303-308_1 | 1DVU | P02766 |
| 4GRR_AVR_B_201 | 0.6528 | 303-308_2 | 4GRR | P14174 |
| 2X7D_EGB_A_1369 | 0.7186 | 303-308_3 | 2X7D | P52732 |
| 3HCC_LT3_A_2001 | 0.6144 | 303-308_4 | 3HCC | P11086 |
| 4I3K_1BX_A_505 | 0.7144 | 303-308_5 | 4I3K | O75874 |
| 1A27_EST_A_350 | 0.7968 | 309-314_0 | 1A27 | P14061 |
| 2B1V_458_A_201 | 0.6327 | 309-314_2 | 2B1V | P03372 |
| 3UUC_0D1_A_1 | 0.6776 | 309-314_3 | 3UUC | P03372 |
| 1IOL_EST_A_400 | 0.6461 | 309-314_4 | 1IOL | P14061 |
| 1Q3W_ATU_A_502 | 0.7043 | 315-318_1 | 1Q3W | P49841 |
| 1XAN_HXP_A_1024 | 0.6623 | 315-318_2 | 1XAN | P00390 |
| 1FDT_EST_A_350 | 0.6718 | 315-318_3 | 1FDT | P14061 |
| 1FDU_EST_A_351 | 0.7940 | 319-324_0 | 1FDU | P14061 |
| 2AM9_TES_A_1000 | 0.7120 | 319-324_1 | 2AM9 | P10275 |
| 2YLO_TES_A_1920 | 0.7151 | 319-324_2 | 2YLO | P10275 |
| 5DI7_5CQ_A_601 | 0.6825 | 319-324_3 | 5DI7 | P03372 |
| 1DHT_DHT_A_400 | 0.6851 | 319-324_4 | 1DHT | P14061 |
| 2AMA_DHT_A_1001 | 0.6892 | 319-324_5 | 2AMA | P10275 |
| 1DHT_DHT_A_400 | 0.7178 | 325-328_0 | 1DHT | P14061 |
| 2X7D_EGB_A_1369 | 0.6703 | 325-328_1 | 2X7D | P52732 |
| 2PIR_DHT_A_931 | 0.7223 | 329-333_2 | 2PIR | P10275 |
| 2AMB_17H_A_1001 | 0.7159 | 329-333_3 | 2AMB | P10275 |
| 1MX9_NLX_A_1 | 0.6338 | 334-338_1 | 1MX9 | P23141 |
| 1E3G_R18_A_1000 | 0.7584 | 334-338_2 | 1E3G | P10275 |
| 3NHU_M42_A_233 | 0.6869 | 334-338_3 | 3NHU | P16083 |
| 3KM0_AOM_C_1 | 0.6625 | 334-338_4 | 3KM0 | P14061 |
| 5DXQ_5HZ_A_900 | 0.6854 | 339-342_1 | 5DXQ | P03372 |
| 1HNN_SKF_A_3001 | 0.7146 | 347-349_2 | 1HNN | P11086 |

**Note:** Database represent: PdbID_LigandID_Chain_NumberOfResidues

**Table S4: Docking based secondary TR screening result: Best ring –target pairs**

| **Ring-ID** | ***Row ID** | **No. of Target** | **UniprotID** |
| --- | --- | --- | --- |
| 334-338_4 | 8 | 16 | Q13093, P51449, P34913, P05093, P20813, P52732, P9WGR1, O43318, P53350, P37173, P36897, Q04771, P04150, O43353, P15559, P28482 |
| 144-147_0 | 4 | 12 | P23141, P01215, P00390, P11021, O14684, Q13526, Q16539, O75874, P02766, P37231, O15164, P50053 |
| 329-333_2 | 2 | 6 | P10275, P14061, P03372, O76083, P10828, P68871 |
| 144-147_2 | 47 | 6 | P56221, P14618, P55201, Q03181, Q99683, Q16875 |
| 334-338_3 | 48 | 6 | P29474, P10275, P36544, P14920, P11086, P42262 |
| 292-296_0 | 63 | 6 | P49327, P16083, Q14289, P11387, Q07343, P09211 |
| 315-318_1 | 18 | 4 | P07900, P15289, Q16853, P06280 |
| 144-147_1 | 21 | 4 | P49841, O00763, Q9H8M2, Q02127 |
| 315-318_3 | 52 | 4 | Q13627,Q86U86, P50579, P08684 |
| **339-342_1** | **1** | **3** | **P10826, P15090, Q13133** |
| 257-262_4 | 43 | 3 | P04406, P04637, P33316 |
| 251-256_0 | 60 | 3 | Q15119, P35790, P02753 |
| 319-324_5 | 11 | 2 | P28845, P05091 |
| 257-262_5 | 40 | 2 | P55072, P29320, |
| 269-273_4 | 45 | 2 | Q9UIF8, P53779 |
| 303-308_4 | 24 | 1 | O76074 |
| 319-324_2 | 6 | 1 | O00408 |
| 334-338_2 | 9 | 1 | Q9NUW8 |
| 319-324_3 | 14 | 1 | Q9NZJ5 |
| 325-328_1 | 20 | 1 | P53582 |
| 138-143_3 | 22 | 1 | P14555 |
| 132-137_2 | 25 | 1 | P19793 |
| 286-291_1 | 29 | 1 | O60885 |
| 269-273_0 | 32 | 1 | P22736 |
| 274-279_4 | 35 | 1 | P25440 |
| 132-137_0 | 42 | 1 | P11509 |
| 125-131_5 | 54 | 1 | Q92793 |
| 242-250_3 | 61 | 1 | P05362 |
| 117-124_7 | 66 | 1 | O75460 |
| 309-314_3 | 67 | 1 | Q07912 |
| 286-291_5 | 26 | 1 | P55055 |
| 263-268_4 | 33 | 1 | P15917 |
| 132-137_1 | 37 | 1 | P14174 |

*Row ID is assigned in TR matrix (Ring-Ranking sheet) supplementary-file 2. The TR matrix is identical to the heatmap (Figure3) after vertical flip.

**5. Materials and Methods:**

**5.1. Synthetic chemistry:**

***5.1.1 General procedure of chemistry:***

All reactions were performed under nitrogen atmosphere (anhydrous conditions). All of the reagents were purchased from Sigma Aldrich, TCI, Alfa Aeser with a purity of 95% or higher and used without further purification. All of the experimental instruments used in the reaction were thoroughly washed and then dried in a dry oven at 70° C. Anhydrous tetrahydrofuran (THF), toluene, diethyl ether (Et2O), tert-butyl methyl ether (TBME) and dimethylformamide (DMF) was purchased from Sigma Aldrich. Iridium catalysts and organolithium catalysts were stored in a refrigerator at -10° C under dark conditions. The reaction pattern was confirmed by UV irradiation, CAM stain (Cerium Ammonium Molybdate stain), PMA stain (Phospho molybdic acid stain) and Ninhydrin Stain on Silica TLC plate (60 F-254). BRUKER Ascend NMR instruments used for 1H-NMR (600 MHz) and 13C-NMR (150 MHz) spectra in CDCl3 solvent with regard to tetramethylsilane as the reference for chemical shift values. The chemical shifts (δ) values were expressed in parts per million (ppm) and coupling constants (J) in hertz (Hz).

**6. Results:**

***6.1. Synthesis of cyproheptadine derivatives and reagent library:***

The selected compounds (6a-f and 8a-b) (**Table 1**) were synthesized starting from 4-(5H-dibenzo[a,d][7]annulen-5-ylidene)piperidine. The 4-(5H-dibenzo[a,d][7]annulen- 5-ylidene)piperidine was obtained from commercially available cyproheptadine hydrochloride sesquihydrate (**1**), which was transformed into cyproheptadine (**2**) using 10% NaOH in ethanol. The demethylation was afforded by converting **2** into ethyl carboxylate derivative (**3**) followed by hydrolysis using KOH in ethanol to obtain 4-(5H-dibenzo[a,d][7]annulen-5-ylidene)piperidine (**4**) **(Fig. S1)**. The obtained **4** is further N-arylated using various functional reagent library of methyl bromo benzoate derivative to obtain the target compound. **Fig. S2** outlined the reactions and condition for synthesis of target compound. Moreover, due to unavailability of some functional reagent library, it was prepared by performing the nitration of bromo benzoic acid (**see section of 5.7**).

***6.2. Procedure for synthesis of starting compound (4):***

 **Fig. S1. General scheme for synthesis of 4-(5H-dibenzo[a,d][7]annulen-5-ylidene)piperidine (4).**

**4-(5H-dibenzo[a,d][7]annulen-5-ylidene)-1-methylpiperidine (2)**

To cyproheptadine hydrochloride sesquihydrate (**1**) (5500 mg, 17 mmol, 1 equiv.) in ethyl acetate (55 mL) aq. NaOH (2.2 g dissolved in 22 mL H2O, 55 mmol, 3.2 equiv) was added. The mixture was stirred at room temperature for 10 minute. Product was extracted with dichloromethane 3 times. The combined organic layer was dried over sodium sulphate, filtered and concentrated under reduced pressure to give a crude product as a white solid. (4640 mg, 95%). ^1^H NMR (600 MHz, CDCl3) δ 7.33 (m, 6H), 7.25 (m, 4H), 7.18 (m, 3H), 6.92 (s, 3H), 4.11 (q, *J* = 7.1 Hz, 3H), 3.64 (s, 3H), 3.07 (ddd, *J* = 12.9, 9.1, 3.8 Hz, 3H), 2.19 (m, 6H), 1.23 (t, *J* = 7.1 Hz, 5H). ^13^C NMR (151 MHz, CDCl3) δ 139.17, 135.25, 134.78, 133.36, 130.99, 128.50, 128.17, 127.74, 126.23, 57.26, 46.09, 30.94, 30.18.

**Ethyl 4-(5H-dibenzo[a,d][7]annulen-5-ylidene)piperidine-1-carboxylate (3)**

4-(5H-dibenzo[a,d][7]annulen-5-ylidene)-1-methylpiperidine (4500 mg) was taken with 90 mL toluene. Ethylchloroformate (4.5 mL, 47 mmol, 3 equiv.) was added to the reaction mixture and refluxed for 3 hour. The progress of the reaction was monitored by TLC using 10% ethyl acetate in hexane as a mobile phase. The product was washed with 50 mL water 3 times. The organic layer was dried over sodium sulphate, filtered and concentrated under reduced pressure to give a crude product as a white solid. (4330 mg, 80 %). ^1^H NMR (600 MHz, CDCl3) δ 7.35 – 7.30 (m, 6H), 7.27 – 7.22 (m, 4H), 7.20 – 7.16 (m, 3H), 6.92 (s, 3H), 4.11 (q, *J* = 7.1 Hz, 3H), 3.64 (s, 3H), 3.07 (ddd, *J* = 12.9, 9.1, 3.8 Hz, 3H), 2.34 – 2.06 (m, 6H), 1.23 (t, *J* = 7.1 Hz, 5H). ^13^C NMR (151 MHz, CDCl3) δ 155.46, 138.74, 134.80, 134.68, 134.46, 130.95, 128.27, 128.23, 127.87, 126.43, 61.23, 45.26, 14.69.

**4-(5H-dibenzo[a,d][7]annulen-5-ylidene)piperidine (4)**

To 4-(5H-dibenzo[a,d][7]annulen-5-ylidene)piperidine-1-carboxylate (**3**) (4600 mg, 13.3 mmol, 1 equiv.) in ethanol (82 mL) aq. KOH (11.2 g dissolved in 20 mL water, 200 mmol, 15 equiv) was added. The mixture was refluxed for 18 hour. The progress of the reaction was monitored by TLC using 10% ethyl acetate in hexane as a mobile phase. The product was washed with 50 mL water 3 times. The organic layer was dried over sodium sulphate and filtered and concentrated under reduced pressure to give a crude product as a white solid (3200 mg, 90%). ^1^H NMR (600 MHz, CDCl3) δ 7.32 (m, 6H), 7.21 (dtd, *J* = 11.3, 8.1, 4.1 Hz, 6H), 6.92 (s, 3H), 2.89 (m, 3H), 2.66 (ddd, *J* = 12.0, 8.5, 3.6 Hz, 3H), 2.26 (ddd, *J* = 16.0, 7.9, 3.7 Hz, 3H), 2.09 (ddd, *J* = 13.5, 6.1, 3.6 Hz, 3H), 1.54 (s, 2H). ^13^C NMR (151 MHz, CDCl3) δ 139.16, 136.42, 134.80, 133.03, 130.99, 128.47, 128.18, 127.73, 126.18, 48.61, 31.99.

***6.3. General Procedure for the Synthesis of 5 (a-g):***

To 4-(5H-dibenzo[a,d][7]annulen-5-ylidene)piperidine (**4**) (100 mg, 0.37 mmol, 1 equiv.) in anhydrous toluene (10 mL) was added the aryl bromide (1 equiv.), sodium tert-butoxide (1 equiv.), 2-Dicyclohexylphosphino-2′,6′-dimethoxybiphenyl (0.3 equiv.), Tris (dibenzylideneacetone) dipalladium (0.05 equiv.). The mixture was refluxed for 12 hour. The residue was extracted with dichloromethane 3 times. The combined organic layer was dried over sodium sulphate and filtered and concentrated under reduced pressure to give a crude product. The crude product was purified by column chromatography (10% ethyl acetate in hexanes).

**Fig. S2.** General scheme for synthesis of target compounds 6a-f

**Methyl 5-(4-(5H-dibenzo [a, d] [7] annulen-5-ylidene) piperidin-1-yl)-2-methylbenzoate (5a)**

^1^H NMR (600 MHz, CDCl3) δ 7.80 (m, 1H), 7.34 (m, 2H), 7.25 (m, 3H), 6.94 (s, 1H), 6.90 (d, *J* = 8.4 Hz, 1H), 3.86 (s, 1H), 3.02 (m, 1H), 2.68 (m, 1H), 2.49 (ddd, *J* = 13.3, 9.1, 4.0 Hz, 1H), 2.31 (m, 3H). ^13^C NMR (151 MHz, CDCl3) δ 167.23, 156.16, 138.99, 134.86, 134.79, 134.15, 132.49, 131.57, 131.00, 128.44, 128.30, 128.25, 127.80, 126.34, 123.67, 118.33, 103.09, 55.26, 53.12, 51.80, 30.60, 29.70, 18.28. White solid (47 mg, 32% yield).

**Methyl 4-(4-(5H-dibenzo[a,d][7]annulen-5-ylidene)piperidin-1-yl)-2-methylbenzoate (5b)**

^1^H NMR (600 MHz, CDCl3) δ 7.58 (s, 1H), 7.34 (dd, *J* = 11.1, 4.4 Hz, 4H), 7.26 (m, 4H), 7.21 (m, 2H), 7.11 (d, *J* = 8.5 Hz, 1H), 7.01 (dd, *J* = 8.4, 2.7 Hz, 1H), 6.92 (s, 2H), 3.34 (m, 2H), 2.95 (ddd, *J* = 12.1, 9.0, 3.5 Hz, 2H), 2.53 (d, *J* = 9.5 Hz, 3H), 2.47 (ddd, *J* = 13.3, 8.9, 4.1 Hz, 2H), 2.27 (ddd, *J* = 13.7, 5.9, 3.7 Hz, 2H). ^13^C NMR (151 MHz, CDCl3) δ 156.85, 138.97, 134.79, 134.77, 134.22, 133.14, 131.52, 131.01, 129.05, 128.43, 128.25, 127.81, 126.36, 118.34, 53.03, 30.57, 29.70, 18.36. White solid (10 mg, 16% yield).

**Methyl 4-(4-(5H-dibenzo[a,d][7]annulen-5-ylidene)piperidin-1-yl)benzoate (5c)**

^1^H NMR (600 MHz, CDCl3) δ 7.88 (m, 2H), 7.34 (m, 2H), 7.26 (m, 2H), 7.20 (dt, *J* = 9.7, 4.6 Hz, 1H), 6.92 (s, 1H), 6.80 (m, 1H), 3.85 (s, 1H), 3.53 (m, 1H), 3.08 (ddd, *J* = 12.7, 9.2, 3.7 Hz, 1H), 2.45 (ddd, *J* = 13.7, 9.2, 4.3 Hz, 1H), 2.26 (ddd, *J* = 14.1, 5.7, 3.7 Hz, 1H), 1.55 (s, 1H). ^13^C NMR (151 MHz, CDCl3) δ 167.18, 153.54, 138.78, 134.74, 134.54, 134.47, 131.27, 130.99, 128.32, 128.30, 127.89, 126.46, 118.83, 113.40, 51.59, 49.10, 29.33. White solid (51 mg, 33% yield).

**Methyl 4-(4-(5H-dibenzo[a,d][7]annulen-5-ylidene)piperidin-1-yl)-3-nitrobenzoate (5d)**

^1^H NMR (600 MHz, CDCl3) δ 8.39 (d, *J* = 2.1 Hz, 1H), 7.98 (dd, *J* = 8.8, 2.1 Hz, 1H), 7.32 (dd, *J* = 10.8, 4.4 Hz, 5H), 7.24 (m, 3H), 7.19 (dd, *J* = 7.1, 2.1 Hz, 2H), 6.96 (m, 1H), 6.90 (s, 2H), 3.87 (s, 3H), 3.23 (m, 2H), 2.91 (ddd, *J* = 12.5, 9.5, 3.4 Hz, 2H), 2.50 (ddd, *J* = 13.8, 9.4, 4.2 Hz, 2H), 2.28 (ddd, *J* = 14.2, 5.4, 3.6 Hz, 2H). ^13^C NMR (151 MHz, CDCl3) δ 165.37, 148.62, 139.47, 138.53, 135.30, 134.65, 134.11, 133.10, 130.95, 128.70, 128.33, 128.20, 127.94, 126.56, 120.45, 119.12, 52.21, 51.84, 31.59, 29.68, 22.65, 14.13. Yellow solid (91 mg, 87% yield).

**Methyl 3-(4-(5H-dibenzo[a,d][7]annulen-5-ylidene)piperidin-1-yl)benzoate (5e)**

^1^H NMR (600 MHz, CDCl3) δ 7.55 (m, 1H), 7.46 (d, *J* = 7.7 Hz, 1H), 7.34 (dd, *J* = 11.0, 4.5 Hz, 2H), 7.26 (m, 2H), 7.22 (m, 1H), 7.07 (m, 1H), 6.92 (s, 1H), 3.88 (s, 3H), 3.40 (m, 1H), 2.99 (ddd, *J* = 12.3, 9.1, 3.6 Hz, 1H), 2.47 (ddd, *J* = 13.4, 9.0, 4.2 Hz, 1H), 2.28 (ddd, *J* = 13.8, 5.8, 3.7 Hz, 1H), 2.17 (d, *J* = 3.1 Hz, 3H). ^13^C NMR (151 MHz, CDCl3) δ 206.99, 167.46, 151.02, 138.90, 134.93, 134.77, 134.11, 131.00, 130.89, 129.05, 128.37, 128.28, 127.85, 126.39, 120.67, 120.12, 116.86, 52.05, 50.93, 30.94, 29.63. White solid (66 mg, 90% yield).

**Methyl 2-(4-(5H-dibenzo[a,d][7]annulen-5-ylidene)piperidin-1-yl)benzoate (5f)**

^1^H NMR (600 MHz, CDCl3) δ 7.55 (m, 1H), 7.46 (d, *J* = 7.6 Hz, 1H), 7.34 (dd, *J* = 11.1, 4.4 Hz, 4H), 7.26 (m, 4H), 7.21 (m, 2H), 7.06 (dd, *J* = 8.2, 2.1 Hz, 1H), 6.92 (s, 2H), 5.29 (s, 1H), 3.88 (s, 3H), 3.40 (m, 2H), 2.99 (ddd, *J* = 12.3, 9.1, 3.6 Hz, 2H), 2.47 (ddd, *J* = 13.4, 9.0, 4.2 Hz, 2H), 2.28 (ddd, *J* = 13.8, 5.8, 3.7 Hz, 2H). ^13^C NMR (151 MHz, CDCl3) δ 167.44, 151.01, 138.89, 134.92, 134.77, 134.10, 130.99, 130.88, 129.04, 128.37, 128.27, 127.84, 126.38, 120.66, 120.12, 116.85, 52.04, 50.91, 29.62. White solid (18 mg, 26% yield).

**Methyl 3-(4-(5H-dibenzo[a,d][7]annulen-5-ylidene)piperidin-1-yl)-5-nitrobenzoate (5g)**

^1^H NMR (600 MHz, CDCl3) δ 7.98 (d, *J* = 10.1 Hz, 1H), 7.34 (m, 5H), 7.27 (dd, *J* = 12.0, 4.5 Hz, 3H), 7.19 (dd, *J* = 5.8, 2.8 Hz, 2H), 6.92 (s, 2H), 6.73 (m, 2H), 3.91 (s, 3H), 3.60 (m, 2H), 3.14 (ddd, *J* = 12.9, 9.5, 3.6 Hz, 2H), 2.46 (ddd, *J* = 14.0, 9.4, 4.4 Hz, 2H), 2.28 (m, 2H). ^13^C NMR (151 MHz, CDCl3) δ 168.02, 153.13, 138.39, 135.62, 134.91, 134.60, 132.85, 132.12, 130.94, 128.40, 128.10, 127.98, 127.11, 126.64, 112.92, 111.70, 53.22, 48.15, 29.69, 29.07. Yellow solid (50 mg, 24% yield).

***6.4. General Procedure for the Synthesis of 6 (a-g):***

To compound **5(a-g)** (1equiv.) in ethanol (10 mL), KOH (22 equiv.) was added. The mixture was refluxed for 12 hour. The residue was extracted with dichloromethane 3 times. The combined organic layer was dried over sodium sulphate and filtered and concentrated under reduced pressure to give a crude product. The filtrate was purified by column chromatography (10% ethyl acetate and 1% acetic acid in hexanes).

**5-(4-(5H-dibenzo[a,d][7]annulen-5-ylidene)piperidin-1-yl)-2-methylbenzoic acid (6a)**

^1^H NMR (600 MHz, CDCl3) δ 7.85 (dd, *J* = 13.4, 5.0 Hz, 2H), 7.34 (ddd, *J* = 6.6, 3.8, 1.2 Hz, 4H), 7.25 (m, 6H), 6.94 (s, 2H), 6.91 (d, *J* = 8.4 Hz, 1H), 3.05 (m, 2H), 2.70 (m, 2H), 2.50 (ddd, *J* = 13.3, 9.2, 4.0 Hz, 2H), 2.31 (m, 5H). ^13^C NMR (151 MHz, CDCl3) δ 156.85, 138.97, 134.79, 134.77, 134.22, 133.14, 131.52, 131.01, 129.05, 128.43, 128.25, 127.81, 126.36, 118.34, 53.03, 30.57, 29.70, 18.36. White solid (25 mg, 86% yield).

**4-(4-(5H-dibenzo[a,d][7]annulen-5-ylidene)piperidin-1-yl)-2-methylbenzoic acid (6b)**

^1^H NMR (600 MHz, CDCl3) δ 7.56 (d, *J* = 2.5 Hz, 1H), 7.34 (dd, *J* = 11.1, 4.4 Hz, 4H), 7.26 (m, 7H), 7.22 (dd, *J* = 8.1, 1.2 Hz, 2H), 7.11 (d, *J* = 8.5 Hz, 1H), 7.01 (dd, *J* = 8.4, 2.8 Hz, 1H), 6.92 (s, 2H), 3.34 (m, 2H), 2.95 (ddd, *J* = 12.2, 9.0, 3.6 Hz, 2H), 2.52 (s, 3H), 2.47 (ddd, *J* = 13.3, 8.9, 4.2 Hz, 2H), 2.36 (s, 1H), 2.28 (ddd, *J* = 13.7, 5.9, 3.6 Hz, 2H). ^13^C NMR (151 MHz, CDCl3) δ 138.93, 135.00, 134.78, 132.55, 131.73, 131.00, 128.39, 128.27, 127.84, 126.37, 121.40, 118.97, 51.42, 29.67, 21.06. White solid (40 mg, 86% yield).

**4-(4-(5H-dibenzo[a,d][7]annulen-5-ylidene)piperidin-1-yl)benzoic acid (6c)**

^1^H NMR (600 MHz, CDCl3) δ 7.93 (m, 2H), 7.34 (m, 2H), 7.26 (m, 3H), 7.21 (m, 1H), 6.93 (s, 1H), 6.81 (d, *J* = 9.1 Hz, 1H), 5.30 (s, 1H), 3.57 (m, 1H), 3.10 (ddd, *J* = 12.7, 9.3, 3.7 Hz, 1H), 2.46 (ddd, *J* = 13.8, 9.2, 4.3 Hz, 1H), 2.27 (ddd, *J* = 14.8, 5.7, 3.8 Hz, 1H). ^13^C NMR (151 MHz, CDCl3) δ 170.78, 153.95, 138.75, 134.73, 134.68, 134.29, 132.06, 130.99, 128.33, 128.28, 127.90, 126.48, 113.16, 48.84, 29.29, 0.00. White solid (45 mg, 90% yield).

**4-(4-(5H-dibenzo[a,d][7]annulen-5-ylidene)piperidin-1-yl)-3-nitrobenzoic acid (6d)**

^1^H NMR (600 MHz, CDCl3) δ 8.47 (d, *J* = 2.1 Hz, 1H), 8.03 (dd, *J* = 8.9, 2.1 Hz, 1H), 7.34 (m, 5H), 7.26 (m, 3H), 7.21 (m, 2H), 6.99 (d, *J* = 9.0 Hz, 1H), 6.92 (s, 2H), 3.28 (m, 2H), 2.96 (ddd, *J* = 12.6, 9.5, 3.5 Hz, 2H), 2.53 (ddd, *J* = 13.8, 9.4, 4.2 Hz, 2H), 2.31 (ddd, *J* = 14.2, 5.3, 3.6 Hz, 2H). ^13^C NMR (151 MHz, CDCl3) δ 170.01, 149.04, 139.21, 138.49, 135.45, 134.64, 134.55, 132.89, 130.95, 129.59, 128.35, 128.17, 127.97, 126.59, 119.13, 118.97, 51.67, 40.47, 29.70, 29.61, 20.76. Yellow solid (16 mg, 54% yield).

**3-(4-(5H-dibenzo[a,d][7]annulen-5-ylidene)piperidin-1-yl)benzoic acid (6e)**

^1^H NMR (600 MHz, CDCl3) δ 7.60 (s, 1H), 7.54 (d, *J* = 7.6 Hz, 1H), 7.33 (m, 6H), 7.25 (dd, *J* = 6.0, 2.2 Hz, 2H), 7.22 (d, *J* = 7.3 Hz, 2H), 7.12 (dd, *J* = 8.2, 2.2 Hz, 1H), 6.92 (s, 2H), 3.41 (m, 2H), 3.00 (m, 2H), 2.48 (ddd, *J* = 13.3, 9.0, 4.1 Hz, 2H), 2.28 (ddd, *J* = 13.7, 5.6, 3.7 Hz, 2H). ^13^C NMR (151 MHz, CDCl3) δ 172.11, 151.04, 138.87, 134.82, 134.77, 134.18, 131.00, 130.03, 129.17, 128.37, 128.28, 127.86, 126.40, 121.54, 120.81, 117.33, 50.92, 31.59, 29.57, 22.66, 14.13. White solid (30 mg, 94% yield).

**2-(4-(5H-dibenzo[a,d][7]annulen-5-ylidene)piperidin-1-yl)benzoic acid (6f)**

^1^H NMR (600 MHz, CDCl3) δ 7.61 (s, 1H), 7.54 (d, *J* = 7.6 Hz, 1H), 7.33 (m, 3H), 7.25 (m, 1H), 7.21 (m, 1H), 7.12 (m, 1H), 6.92 (s, 1H), 3.41 (m, 2H), 3.00 (ddd, *J* = 12.2, 9.1, 3.6 Hz, 1H), 2.48 (ddd, *J* = 13.3, 9.0, 4.1 Hz, 1H), 2.29 (ddd, *J* = 13.7, 5.7, 3.7 Hz, 1H). ^13^C NMR (151 MHz, CDCl3) δ 172.09, 151.03, 138.87, 134.81, 134.76, 134.18, 131.00, 130.03, 129.17, 128.36, 128.28, 127.86, 126.40, 121.53, 120.81, 117.34, 50.92, 29.70, 29.56. White solid (27 mg, 94% yield).

**3-(4-(5H-dibenzo[a,d][7]annulen-5-ylidene)piperidin-1-yl)-5-nitrobenzoic acid (6g)**

1H NMR (600 MHz, CDCl3) δ 7.98 (d, *J* = 9.3 Hz, 1H), 7.35 (t, *J* = 6.5 Hz, 2H), 7.26 (m, 4H), 7.20 (d, *J* = 7.3 Hz, 1H), 6.93 (s, 1H), 6.82 (d, *J* = 1.9 Hz, 1H), 6.74 (d, *J* = 9.1 Hz, 1H), 3.62 (dd, *J* = 11.7, 5.7 Hz, 2H), 3.15 (m, 1H), 2.47 (ddd, *J* = 13.8, 9.3, 4.2 Hz, 1H), 2.29 (m, 1H). 13C NMR (151 MHz, CDCl3) δ 154.15, 138.44, 134.62, 130.96, 128.41, 128.13, 128.00, 126.64, 111.89, 105.21, 102.06, 48.24, 29.71, 29.10. Yellow solid (5 mg, 50% yield).

***6.5. General Procedure for the Synthesis of 7(a-b):***

**Fig. S3.** General scheme for synthesis of target compounds 7 and 8

To the compound containing nitro functional group (**5d, 5g**) (1 equiv.) in ethanol (0.5 mL) was added iron powder (3 equiv.) and aq. HCl 16 % v/v (1 equiv.). The mixture was stirred at 80°C for 12 hour. The residue was concentrated and filtered through a funnel packed with celite. The residue was extracted with dichloromethane 3 times. The combined organic layer was dried over sodium sulphate and filtered and concentrated under reduced pressure to give a crude product. The crude product was purified by column chromatography (10% ethyl acetate in hexanes).

**Methyl 4-(4-(5H-dibenzo[a,d][7]annulen-5-ylidene)piperidin-1-yl)-3-aminobenzoate (7a)**

^1^H NMR (600 MHz, CDCl3) δ 7.39 (dd, *J* = 8.2, 1.9 Hz, 1H), 7.37 (d, *J* = 1.9 Hz, 1H), 7.34 (ddd, *J* = 6.6, 3.6, 2.0 Hz, 5H), 7.25 (m, 8H), 6.94 (s, 2H), 6.90 (d, *J* = 8.2 Hz, 1H), 3.96 (s, 2H), 3.85 (s, 3H), 3.01 (m, 2H), 2.66 (s, 2H), 2.47 (ddd, *J* = 13.2, 9.0, 3.9 Hz, 2H), 2.30 (dd, *J* = 10.1, 4.8 Hz, 2H). ^13^C NMR (151 MHz, CDCl3) δ 168.70, 167.29, 150.03, 143.88, 140.81, 138.95, 134.77, 134.71, 134.24, 131.00, 128.41, 128.25, 127.81, 126.36, 125.50, 120.44, 119.10, 115.86, 102.48, 52.44, 51.85, 30.83. Yellow solid (20 mg, 45% yield).

**Methyl 3-(4-(5H-dibenzo[a,d][7]annulen-5-ylidene)piperidin-1-yl)-5-aminobenzoate (7b)**

^1^H NMR (600 MHz, CDCl3) δ 7.34 (m, 5H), 7.24 (m, 4H), 7.03 (dd, *J* = 8.8, 2.5 Hz, 1H), 6.92 (s, 2H), 6.60 (d, *J* = 8.9 Hz, 1H), 5.41 (s, 2H), 3.84 (s, 3H), 3.13 (m, 2H), 2.75 (m, 2H), 2.47 (ddd, *J* = 12.9, 9.0, 3.8 Hz, 2H), 2.29 (ddd, *J* = 13.5, 5.4, 3.5 Hz, 2H). ^13^C NMR (151 MHz, CDCl3) δ 168.47, 145.16, 142.51, 139.02, 135.19, 134.79, 133.73, 131.00, 128.46, 128.22, 127.79, 126.96, 126.31, 118.61, 117.86, 110.77, 60.40, 53.38, 51.49, 30.18, 29.70, 14.20, 14.13. Yellow solid (20 mg, 45% yield).

***6.6. General Procedure for the Synthesis of 8 (a-b):***

To compound **7(a-b)** (1equiv.) in ethanol (10 mL), KOH (22 equiv.) was added. The mixture was refluxed for 12 hour. The residue was extracted with dichloromethane 3 times. The combined organic layer was dried over sodium sulphate and filtered and concentrated under reduced pressure to give a crude product. The filtrate was purified by column chromatography (10% ethyl acetate and 1% acetic acid in hexanes).

**4-(4-(5H-dibenzo[a,d][7]annulen-5-ylidene)piperidin-1-yl)-3-aminobenzoic acid (8a)**

^1^H NMR (600 MHz, CDCl3) δ 7.46 (d, *J* = 8.3 Hz, 1H), 7.42 (s, 1H), 7.34 (m, 2H), 7.25 (m, 2H), 6.93 (d, *J* = 11.1 Hz, 3H), 3.11 (s, 1H), 2.70 (s, 1H), 2.57 (s, 1H), 2.35 (d, *J* = 10.0 Hz, 1H). ^13^C NMR (151 MHz, CDCl3) δ 140.76, 138.76, 134.73, 130.99, 128.35, 128.28, 127.87, 126.45, 121.23, 119.19, 52.62, 29.70. Yellow solid (18 mg, 90% yield).

**3-(4-(5H-dibenzo[a,d][7]annulen-5-ylidene)piperidin-1-yl)-5-aminobenzoic acid (8b)**

^1^H NMR (600 MHz, CDCl3) δ 7.43 (m, 1H), 7.33 (m, 6H), 7.23 (m, 4H), 7.12 (d, *J* = 6.7 Hz, 1H), 6.93 (d, *J* = 6.8 Hz, 2H), 6.62 (d, *J* = 8.9 Hz, 1H), 3.16 (m, 2H), 2.79 (t, *J* = 8.6 Hz, 2H), 2.51 (s, 2H), 2.30 (m, 2H). ^13^C NMR (151 MHz, CDCl3) δ 162.09, 138.93, 134.77, 131.00, 128.43, 128.24, 127.83, 126.36, 118.08, 109.30, 29.70, 22.70. Yellow solid (13 mg, 94% yield).

***6.7. Synthesis of reagent library and target compound:***

As outlined in **Fig. S3**, the reagent library was prepared starting from protecting the carboxylic group through methylation using H_2_SO_4_ and MeOH as reagent and solvent. Regardless of the ortho, meta, and para positions of the carboxyl group, the reaction proceeded at a high yield. The HNO_3_ and H_2_SO_4_ were used for nitrification. At first step, both Sphos and palladium catalysts were used 10 times more than the conventional reaction to maintain the anhydrous condition. The obtained substituted Nitro derivatives were reduced to amine using iron, and the reaction proceeded at a high yield regardless of the reagent. Finally, the final product was completed through demethylation using KOH.

**Fig. S3: Synthesis of reagent libraries (substituted aryl halides)**

*6.8. General Procedure for the Synthesis of 9(a-c):*

The substituted bromo benzoic acid was dissolved in methanol, and conc. H_2_SO_4_ (4.6 equiv.) was added dropwise while stirring. The reaction mixture was heated to reﬂux and stirred for 12 hour. The reaction mixture was quenched in a saturated aq NaHCO_3_ solution ensuring neutral to basic pH, and the aqueous phase was extracted with dichloromethane 3 times. The combined organic phases were washed with saturated aq NaCl solution and dried over anhydrous Na_2_SO_4_ and ﬁltered. The solvent was removed under reduced pressure to obtain 8a-8c product.

**Methyl 5-bromo-2-methylbenzoate (9a)**

^1^H NMR (600 MHz, CDCl3) δ 8.04 (d, *J* = 2.2 Hz, 1H), 7.51 (dd, *J* = 8.2, 2.2 Hz, 1H), 7.12 (s, 1H), 3.90 (s, 3H), 2.54 (s, 3H). ^13^C NMR (151 MHz, CDCl3) δ 166.68, 139.17, 134.80, 133.33, 133.28, 131.16, 119.09, 52.10, 21.21. White solid (89 mg, 41% yield).

**Methyl 4-bromobenzoate (9b)**

^1^H NMR (600 MHz, CDCl3) δ 7.90 (m, 2H), 7.58 (m, 2H), 3.92 (s, 3H). ^13^C NMR (151 MHz, CDCl3) δ 166.37, 131.71, 131.11, 129.02, 128.02, 52.28. White solid (5028 mg, 94% yield).

**Methyl 3-bromobenzoate (9c)**

^1^H NMR (600 MHz, CDCl3) δ 8.18 (t, *J* = 1.7 Hz, 1H), 7.97 (m, 1H), 7.68 (ddd, *J* = 8.0, 2.0, 1.1 Hz, 1H), 7.32 (t, *J* = 7.9 Hz, 1H), 3.93 (s, 3H). ^13^C NMR (151 MHz, CDCl3) δ 165.76, 135.87, 132.61, 132.04, 129.94, 128.15, 122.44, 52.43. White solid (4391 mg, 82% yield).

***6.9. General Procedure for the Synthesis of 10(a-d):***

The 60% nitric acid (15 equiv.) and 95% sulfuric acid (30 equiv.) were added to the substituted methyl bromobenzoate at 0° C. The reaction mixture was stirred for 5 hour at 0° C and stirred further for 2 hour at room temperature and then poured over ice. The reaction mixture was quenched in a saturated 10% NH4OH solution ensuring acid to neutral pH. The aqueous phase was extracted with dichloromethane 3 times. The combined organic phases were washed with saturated aq NaCl solution, dried over anhydrous Na2SO4 and filtered. The solvent was removed under reduced pressure to obtain 9a-9d product.

**Methyl 4-bromo-3-nitrobenzoate (10a)**

^1^H NMR (600 MHz, CDCl3) δ 8.47 (m, 1H), 8.07 (dt, *J* = 8.3, 2.0 Hz, 1H), 7.85 (dd, *J* = 8.3, 1.9 Hz, 1H), 3.97 (d, *J* = 1.6 Hz, 3H). ^13^C NMR (151 MHz, CDCl3) δ 164.33, 149.87, 135.44, 133.47, 130.71, 126.48, 119.58, 52.95. White solid (450 mg, 23% yield).

**Methyl 3-bromo-5-nitrobenzoate (10b)**

^1^H NMR (600 MHz, CDCl3) δ 7.86 (d, *J* = 2.1 Hz, 1H), 7.83 (d, *J* = 8.6 Hz, 1H), 7.76 (dd, *J* = 8.6, 2.1 Hz, 1H), 3.94 (s, 3H). ^13^C NMR (151 MHz, CDCl3) δ 164.61, 146.68, 134.66, 132.76, 129.31, 127.84, 125.47, 53.59. White solid (262 mg, 28% yield).

**Methyl 3-bromo-2-nitrobenzoate (10c)**

^1^H NMR (600 MHz, CDCl3) δ 7.86 (d, *J* = 2.1 Hz, 1H), 7.83 (d, *J* = 8.6 Hz, 1H), 7.76 (dd, *J* = 8.6, 2.1 Hz, 1H), 3.94 (s, 3H). ^13^C NMR (151 MHz, CDCl3) δ 162.48, 137.90, 130.89, 130.31, 124.47, 114.38, 53.34. White solid (38 mg, 4% yield).

**Methyl 2-bromo-5-nitrobenzoate (10d)**

^1^H NMR (600 MHz, CDCl3) δ 8.66 (m, 1H), 8.17 (m, 1H), 7.87 (ddd, *J* = 10.3, 6.4, 2.7 Hz, 1H), 4.00 (d, *J* = 3.0 Hz, 3H). ^13^C NMR (151 MHz, CDCl3) δ 164.51, 135.73, 133.03, 129.25, 126.70, 126.63, 126.35, 53.15. White solid (1100 mg, 90% yield).
